# Comparative Sigma Factor-mRNA Levels in *Mycobacterium marinum* under Stress Conditions and during Host Infection

**DOI:** 10.1371/journal.pone.0139823

**Published:** 2015-10-07

**Authors:** B. M. Fredrik Pettersson, Sarbashis Das, Phani Rama Krishna Behra, Heather R. Jordan, Malavika Ramesh, Amrita Mallick, Kate M. Root, Martin N. Cheramie, Irma de la Cruz Melara, Pamela L. C. Small, Santanu Dasgupta, Don G. Ennis, Leif A. Kirsebom

**Affiliations:** 1 Department of Cell and Molecular Biology, Uppsala University Biomedical Centre, Uppsala, Sweden; 2 Department of Microbiology, University of Tennessee, Knoxville, Tennessee, United States of America; 3 Department of Biology, University of Louisiana, Lafayette, Louisiana, United States of America; Bose Institute, INDIA

## Abstract

We have used RNASeq and qRT-PCR to study mRNA levels for all σ-factors in different *Mycobacterium marinum* strains under various growth and stress conditions. We also studied their levels in *M*. *marinum* from infected fish and mosquito larvae. The annotated σ-factors were expressed and transcripts varied in relation to growth and stress conditions. Some were highly abundant such as *sigA*, *sigB*, *sigC*, *sigD*, *sigE* and *sigH* while others were not. The σ-factor mRNA profiles were similar after heat stress, during infection of fish and mosquito larvae. The similarity also applies to some of the known heat shock genes such as the α-crystallin gene. Therefore, it seems probable that the physiological state of *M*. *marinum* is similar when exposed to these different conditions. Moreover, the mosquito larvae data suggest that this is the state that the fish encounter when infected, at least with respect to σ-factor mRNA levels. Comparative genomic analysis of σ-factor gene localizations in three *M*. *marinum* strains and *Mycobacterium tuberculosis* H37Rv revealed chromosomal rearrangements that changed the localization of especially *sigA*, *sigB*, *sigD*, *sigE*, *sigF* and *sigJ* after the divergence of these two species. This may explain the variation in species-specific expression upon exposure to different growth conditions.

## Introduction

The transcription machinery in bacteria, RNA polymerase, is composed of several subunits referred to as the α-, β-, β'-, σ- and ω-subunits [1] where the accessory σ-factor has a key role during initiation of transcription. Two sigma (σ) factor families have been well characterized in bacteria, the σ^70^ and σ^54^ families. On the basis of phylogenetic relationship, the σ^70^ family can be divided into four groups depending on the presence or absence of specific regions; group 1 σ-factors contain several while group 4 σ-factors possess the least number of regions and the number for those belonging to groups 2 and 3 fall in between. The σ-factors belonging to group 4 are also referred to as the extra cytoplasmatic (ECF) σ-factors [2].

During initiation of transcription the σ-factor mediates promoter recognition and interaction with specific residues in the -10 and -35 regions. The sequences of -10 and -35 vary and different σ-factors have different sequence requirements. As such, the σ-factors have key roles in the regulation of gene expression in response to changes in the growth conditions. The number of σ-factors varies among bacterial species. For example, *Escherichia coli* has seven, while more than 60 have been identified in *Streptomyces coelicolor* [3–5]. Between the *Mycobacterium* spp. the number also varies. *Mycobacterium leprae* has four, *Mycobacterium tuberculosis* has thirteen while the *Mycobacterium marinum* M-strain has 18 σ-factor genes, all of which belong to the σ^70^ family. In *Mycobacterium* spp. the σ-factor A (σ^A^ or SigA, which corresponds to σ^70^ in *E*. *coli*) is referred to as the housekeeping σ-factor and it is essential for survival. The other σ-factors do not seem to be essential for growth [5–10].

The levels of expression and activity of σ-factors depend on the stage of growth and environmental conditions. These changes are well documented for *M*. *tuberculosis* and *Mycobacterium smegmatis* grown under various conditions, including growth inside eukaryotic cells. However, the expression levels for orthologous σ-factors vary in *Mycobacterium* spp., so caution should be taken when comparing the levels of individual σ-factors in different strains (reviewed in [5,8–10]). Moreover, information about the levels of expression and distribution of the full-set of σ-factors is scarce for *Mycobacterium* spp. grown under different conditions. We therefore decided to use the fish pathogen *M*. *marinum*, considered to be a model system for some aspects of *M*. *tuberculosis* virulence [11–13], to study the levels of σ-factor mRNAs in cells grown under various conditions by RNASeq and qRT-PCR. We also analyzed the *M*. *marinum* σ-factor mRNA levels during infection of its natural hosts, the Japanese medaka [12] and when *M*. *marinum* resides in the gastrointestinal tract of the yellow fever mosquito larvae (*Aedes aegypti*) which are ingested by the fish [14–16].

Here we present the mRNA profiles of all the annotated σ-factor genes in the *M*. *marinum* T CCUG 20998 strain as a function of growth conditions. The mRNA levels varied for the individual σ-factors as was expected from data for other *Mycobacterium* spp. (see above). For many of the σ-factor genes, such as *sigG*, *sigJ* and *sigM* the mRNA levels were significantly lower than the highly abundant σ-factor transcripts, *sigA*, *sigB*, *sigC*, *sigD*, *sigE* and *sigH*. In particular, the *sigB* mRNA level was substantially increased as a result of several stress conditions; SigB may therefore be a general stress σ-factor in *M*. *marinum*. Interestingly, the *M*. *marinum* σ-factor mRNA levels were similar whether measured in the fish, the mosquito larvae or after exposure to heat stress. The same was true for mRNA levels of the heat shock genes *hsp*, *dnaK*, *dnaJ* and *clpB*. This raises the interesting possibility that specific genes that are expressed after exposure to heat stress are also expressed in *M*. *marinum* during infection and when residing in mosquito larvae and as such may have an impact on virulence.

## Results

### Identification and gene synteny of σ-factors in *M*. *marinum* CCUG

**Fig 1:**
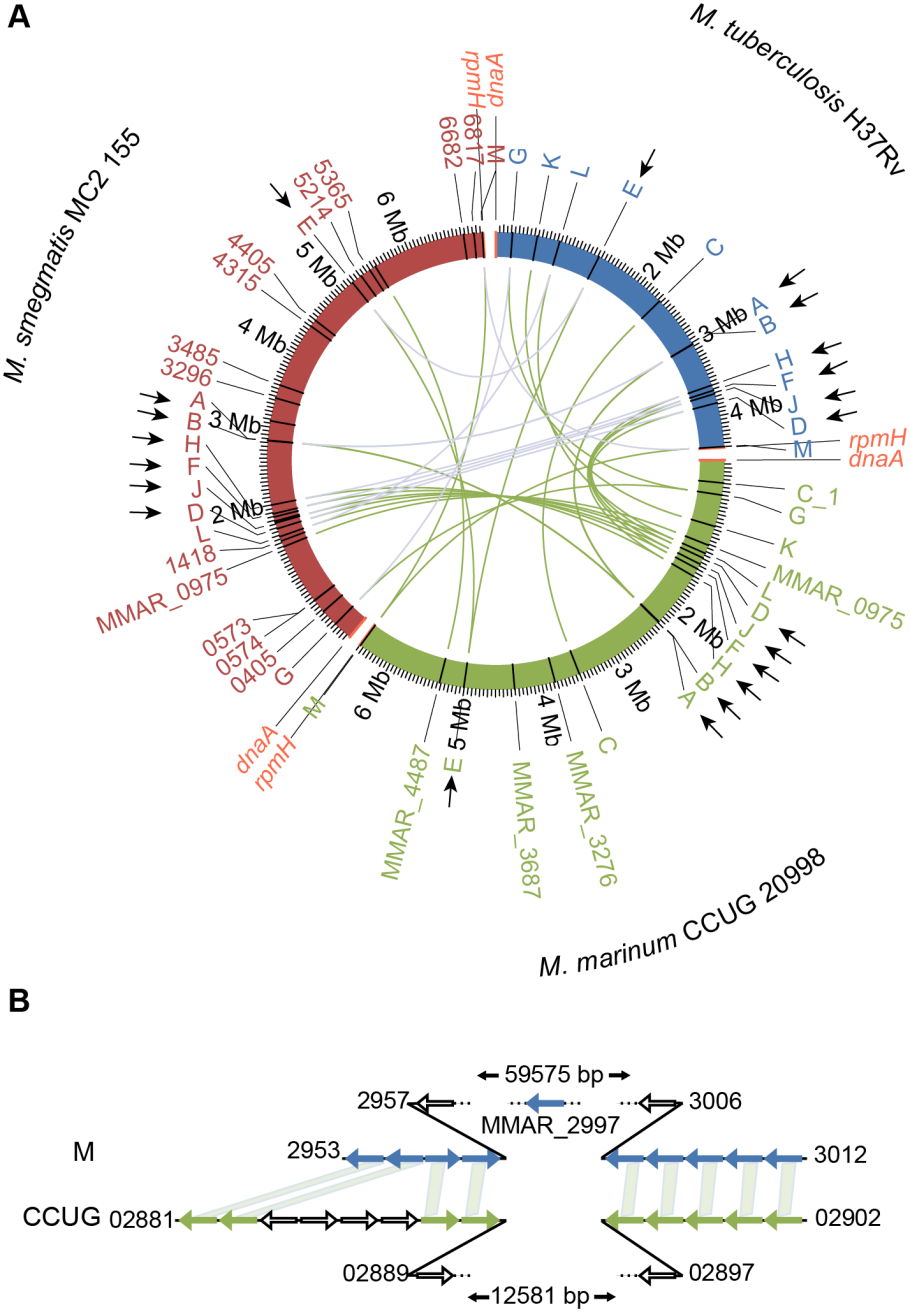
Organization of the σ-factor genes in three *Mycobacterium* species. (A) σ-factor gene organization in *M*. *marinum* CCUG, *M*. *tuberculosis* H37Rv [18], and *M*. *smegmatis* mc^2^155 [19]. The positions of the σ-factor genes on the respective chromosomes are shown in a circular, head-to-tail alignment of the genomes. Homologous σ-factor genes in the three genomes are connected with green lines, while blue lines refer to homologous σ-factor genes in *M*. *tuberculosis* H37Rv and *M*. *smegmatis* mc^2^155. *dnaA* and *rpmH* are positioned at the beginning and end of each genome (marked in bright red). The σ-factor genes marked with black arrows correspond to those that are localized at different positions relative to *dnaA* and *rpmH* in *M*. *tuberculosis* H37Rv relative to the other two *Mycobacterium*. For details see the main text (see also S1 Fig panels A and B). (B) Gene synteny in the *sig2997* region for the *M*. *marinum* CCUG (bottom line marked in green) and M (top line marked in blue) strains. Arrows represent the genes and shaded lines connect homologous genes; open arrows indicate non-homologous genes. The sizes of the regions (in base pairs, bp) and the gene numbers of the first and last genes of the homologous and the non-homologous regions are indicated.

To study the levels of the different σ-factor gene transcripts in *M*. *marinum* T CCUG 20998 we first identified the number of σ-factor genes in this strain. This ATCC strain is hereafter referred to as the *M*. *marinum* "CCUG-strain". It harbors 17 σ-factor genes (Fig 1A; the complete *M*. *marinum* CCUG genome will be published elsewhere). The *M*. *marinum* M strain carries 18 σ-factor genes [17]. Compared to the *M*. *tuberculosis* H37Rv strain, the CCUG-strain has four additional σ-factor genes—MMAR0975, MMAR3276, MMAR3687 and MMAR4487 –but it lacks the σ-factor gene corresponding to the M-strain MMAR2997 σ-factor (Fig 1B). The gene synteny for the σ-factor genes that are present in the CCUG-strain is the same as that for the *M*. *marinum* M-strain (except for MMAR2297, which is missing in CCUG). The same is true for those σ-factor genes in common for *M*. *tuberculosis* H37Rv and the two *M*. *marinum* strains (Fig 1A). When we compared the chromosomal locations of the σ-factor genes relative to *dnaA* and *rpmH* in *M*. *tuberculosis* H37Rv and *M*. *marinum* we noted that the locations for several of the genes were shifted (Fig 1A; *dnaA* and *rpmH*, located near the origins of replication, *oriC*, in both these *Mycobacterium* were used as reference loci [17,18]). In *M*. *tuberculosis* H37Rv *sigD*, *sigF*, *sigH* and *sigJ* are located closer to *rpmH* (Fig 1A) while they are closer to *dnaA* in both the *M*. *marinum* strains (see also S1 Fig panel A). A comparison of the three genomes also revealed that these σ-factor genes are inverted in *M*. *tuberculosis* H37Rv (S1 Fig panel C). The location of *sigE* is also shifted; in *M*. *tuberculosis* H37Rv it is close to *dnaA* but closer to *rpmH* in *M*. *marinum* (Fig 1A). In addition, *sigE* as well as *sigA vs sigB* are inverted in *M*. *marinum* compared to *M*. *tuberculosis* H37Rv (S1 Fig panel C). Together these events indicate that a chromosomal rearrangement occurred after the divergence of these two phylogenetically closely related species leading to a change in the location for some σ-factor genes. The other common σ-factor genes are positioned roughly at the same locations relative to *dnaA* and *rpmH* in both *M*. *marinum* and *M*. *tuberculosis* H37Rv. Interestingly, the chromosomal locations for *sigA*-*M* relative to *dnaA* and *rpmH* are similar for the two *M*. *marinum* strains and the distantly related *M*. *smegmatis* mc^2^155 (excluding *sigC* and *sigK* which are absent in the latter; Fig 1A; see also the discussion). This also applies to the gene synteny (S1 Fig panel D).

### Expression levels of σ-factor genes in cells grown on solid *vs* liquid media

**Fig 2:**
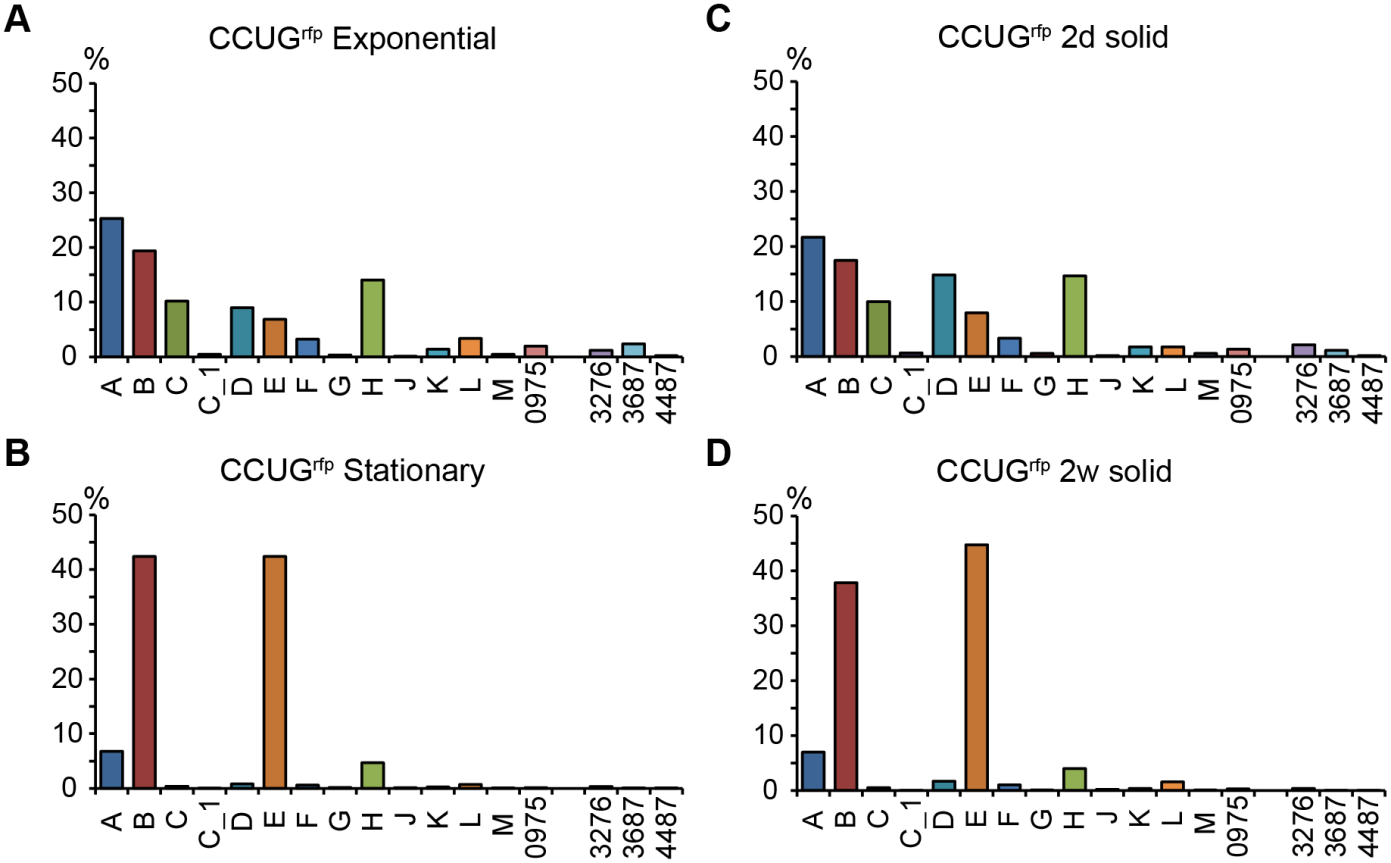
Distribution of σ-factor mRNAs in *M*. *marinum* CCUG^rfp^. (A) Exponential and (B) Stationary cells grown in 7H9 medium. (C) Cultivation on 7H10 for 2 days (d) and (D) 2 weeks (w). The distribution is given as the percentage of reads (from RNASeq data) originating from each individual σ-factor gene relative to the reads originating from all σ-factor genes. The different σ-factors are marked on the x-axis. Note that *sig2997* is absent in the CCUG^rfp^ strain. For details see the main text and Fig 1B.

**Fig 3:**
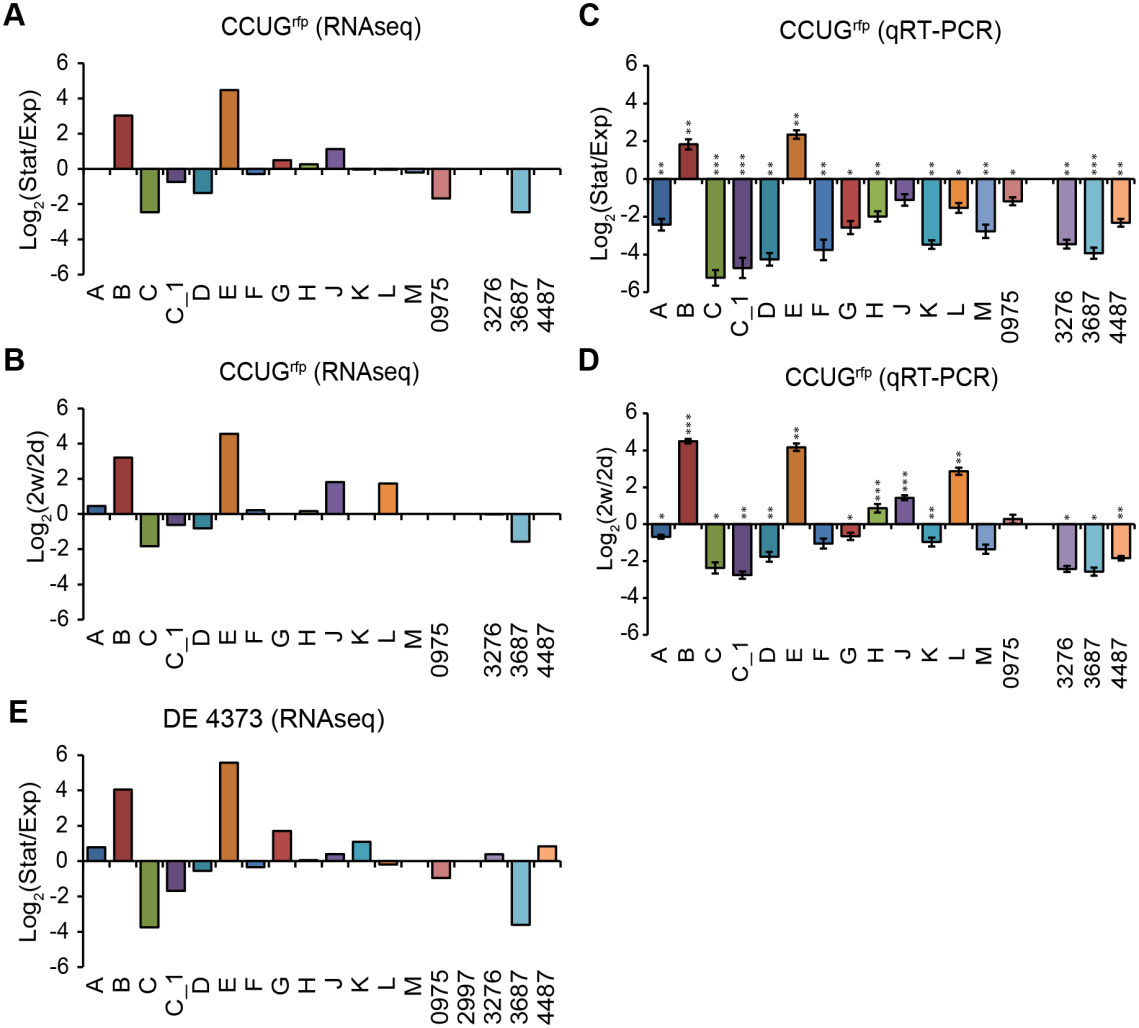
Change of σ-factor mRNA levels as a function of growth phase. (A) Change in σ-factor mRNA levels (RNASeq data) comparing stationary and exponential CCUG^rfp^ cells cultivated in 7H9 medium. (B) Change in σ-factor mRNA levels (RNASeq data) comparing 2 weeks and 2 days old CCUG^rfp^ cells cultivated on 7H10 medium. (C) Change in σ-factor mRNA levels (qRT-PCR data) comparing stationary and exponential CCUG^rfp^ cells cultivated in 7H9 medium. Significant log_2_-fold changes for *sigC*, *sigC_1*, *sigD*, *sig0975* and *sig3687* were 4.1, 2.8, 1.7, 0.2 and 2.5, respectively (p < 0.05). (D) Change in σ-factor mRNA levels (qRT-PCR data) comparing 2 weeks and 2 days old CCUG^rfp^ cells cultivated on 7H10 medium. Significant log_2_-fold changes for *sigB*, *sigE*, *sigJ*, and *sigL* were 4.0, 3.0, 1.2, and 1.8, respectively (p < 0.05), while for *sigC*, *sigC_1*, *sigD*, and *sig 3687* the significant log_2_-fold changes were 1.3, 2.0, 1.2, and 0.5, respectively (p < 0.05). With respect to *sigL* mRNA the significant log_2_-fold change was higher, 1.8 (p < 0.05) *vs* < 0.1 (p < 0.05), when grown on 7H10 medium compared to growth in liquid 7H9 medium (Fig 3C). (E) Change in σ-factor mRNA levels (RNASeq data) comparing exponential and stationary DE4373 cells cultivated in 7H9 medium. The ratios of stationary *vs* exponential phase mRNA levels were plotted on a log_2_ scale so that positive values indicate higher mRNA levels in late stages of growth. The different σ-factors are marked on the x-axis. The RNASeq data (GFOLD values; see Materials and Methods) are based on a single replicate, while the qRT-PCR data are based on at least 6 biological replicates for the exponential and two biological replicates for the stationary samples (except for *sigF* where it is based on three biological replicates). Statistically significant differences are indicated: * p < 0.05; ** p < 0.01; *** p < 0.001.

Like many other *Mycobacterium* spp. *M*. *marinum* can be grown on solid (7H10) media and in liquid (7H9) media (these two media are the same except that 7H9 contains Tween 80 and 7H10 includes agar; see Materials and Methods). We decided to study the σ-factor mRNA levels for cells grown on/ in both media. *M*. *marinum* CCUG/ pDEAM5 cells were grown on solid selective 7H10 medium for two days, one week, and two weeks (*M*. *marinum* CCUG harboring pDEAM5 encoding the red fluorescent protein, *rfp*, gene integrated into the *attB* L5 site on the chromosome, referred to as the "CCUG^rfp^ strain" (Materials and Methods; Pettersson et al., unpublished; see also [20]). Cells were also grown in liquid selective 7H9 medium until the OD_600_ reached 0.5 ± 0.1 (exponential phase) and 4.5 ± 0.5 (stationary phase) (S2 Fig). Cells were harvested and total RNA was extracted and analyzed by RNASeq and qRT-PCR (for details see Materials and Methods). The σ-factor mRNA profiles were calculated in two ways: i) abundance of each σ-factor transcript relative to the sum of all σ-factor transcripts (referred to as σ-factor mRNA distribution; Fig 2); and ii) from the change in σ-factor mRNA levels; for example, the levels detected in stationary *vs*. exponentially growing cells (referred to as change in σ-factor mRNA levels; Fig 3).

Fig 2 shows similar σ-factor distribution profiles at the same growth phase irrespective of the growth media used *i*.*e*., the profiles were the same after growth on solid media for two days and in liquid at exponential phase. This suggests that the cells were growing exponentially for at least two days on solid medium and had reached stationary phase after one week (see S2 Fig). This interpretation is consistent with the high correlation (R-value = 0.948; see also below and Materials and Methods) between the RNASeq data when treating the two days solid medium culture and exponentially growing cells as replicates (data not shown).

The *sigA* transcript was the most abundant σ-factor mRNA during exponential growth phase where it accounted for roughly 20–25% of the total level of σ-factor transcripts (Fig 2). Less abundant transcripts (8–16%) originated from *sigD*, *sigH*, *sigB*, *sigC*, and *sigE* while the levels for *sigF* and *sigL* mRNAs were below 4%. The rest of the σ-factor transcripts such as *sigG*, *sigJ* and *sigM*, were only present in low amounts in exponentially growing cells. In stationary phase (on solid and in liquid media) transcripts from *sigB* and *sigE* constituted roughly 80% of the total amount of σ-factor mRNAs. It appears that the *sigE* transcript became more abundant upon aging (cf. levels after one and two weeks on 7H10 media; Fig 2 and S3 Fig panel A). The increased levels of *sigB* and *sigE* mRNAs in stationary phase were also apparent when we calculated the change in mRNA levels for individual σ-factors in exponentially growing and stationary phase cells (Fig 3A and 3B). There were also changes for other σ-factor mRNAs (see below). Moreover, we noted small differences in the levels of *sigL* and *sig0975* mRNAs comparing solid *vs*. liquid media, which might be related to the way the cells were grown (compare Fig 3A and 3B). However, this is not addressed further in this report.

To confirm and validate these data, we used quantitative real-time PCR (qRT-PCR) to measure the relative σ-factor mRNA levels of samples from cells grown under the same conditions as in the RNASeq analysis. For the liquid 7H9 medium, the qRT-PCR (Fig 3C) revealed that the *sigB* and *sigE* transcripts increased in stationary phase (the increases were significant to 0.7 and 0.9 log_2_-fold, respectively; p < 0.05) while the levels for the other σ-factors were higher in exponentially grown cells (all changes were statistically significant; S1 Table). Both data sets showed that the levels of *sigC*, *sigC_1*, *sigD*, *sig0975* and *sig3687* were higher in cells growing exponentially (Fig 3A and 3C; for statistical significance see legend Fig 3C).

For the solid 7H10 medium (Fig 3D), both the qRT-PCR and the RNASeq data showed increases in the mRNA of *sigB*, *sigE*, *sigJ*, and *sigL*, while *sigC*, *sigC_1*, *sigD*, and *sig3687* mRNAs decreased comparing 2 weeks *vs* 2 days of growth (for statistical significance see legend Fig 3D). The major difference comparing solid and liquid medium was the level of *sigL* mRNA, which in later stages of growth was higher in solid 7H10 but lower for cells grown in liquid 7H9 medium (for statistical significance see legend Fig 3D). These data agree with and thereby validate the RNASeq data for these σ-factors.

Comparison of the RNASeq and qRT-PCR data sets also revealed differences (*e*.*g*., *sigA* and *sigK*; Fig 3A and 3C). These differences may be because the RNASeq σ-factor level were estimated relative to the total rRNA depeleted RNA while the qRT-PCR values were expressed relative to 16S rRNA. Another reason could be RNA degradation (see also [21]).

In conclusion, the σ-factor mRNA levels vary in *M*. *marinum* CCUG in a growth phase dependent manner and the levels are similar, irrespective of growth on solid or in liquid media.

### σ-factor mRNA levels in different *M*. *marinum* strains

To determine if the σ-factor transcript levels are strain specific we generated RNASeq data from exponentially and stationary cell cultures of two other *M*. *marinum* ATCC strains, DE4373 and DE4381 (DE4373 is a derivative of 1218R while DE4381, also referred to as 1218S, is a smooth colony variant of 1218R [22]; see Materials and Methods). The genome organization of the σ-factor genes in the ATCC strain DE4373 is similar to both the CCUG and M strains (S1 Fig panel A), except that *sig2997* is present in the DE4373 and DE4381 strains and absent in CCUG. (The complete genome sequences for DE4373 and DE4381 will be published elsewhere). The transcriptome of the *M*. *marinum* M strain has recently been published [23]. We therefore extracted the σ-factor mRNA data for exponentially and stationary growing cells and included these in our analysis.

The σ-factor mRNA distribution profiles and change in mRNA levels for exponentially and stationary growing DE4373, DE4381 and CCUG cells were similar (compare Figs 2 and 3, S3 and S4 Figs). In keeping with this was the good correlation (R-value = 0.89) comparing the transcriptomes for exponentially grown CCUG^rfp^ and DE4373 (or DE4381) cells, while the R-value was 0.94 comparing DE4373 and DE4381 transcriptome data sets. (The R-values using the stationary transcriptome data sets were slightly lower, data not shown). We did, however, observe small variations for some of the σ-factor mRNAs in the different strains such as *sigG*, *sigL*, *sigM* and *sig3687*; this might be related to the time of sampling. The σ-factor distribution for the M-strain was again similar to that of the CCUG-strain with a few differences such as increased levels of *sigD* and less *sigC* in exponentially growing cells compared to the other three strains (CCUG, DE4373 and DE4381). We also noted that the expression level of *sig2997* was higher in cells at stationary phase than in exponentially growing ones, which was not seen for the DE4373 and DE4381 strains (Fig 3 and S4 Fig; note that the CCUG-strain lacks *sig2997*). These differences were in keeping with that the R-values comparing the transcriptomes for exponential (or stationary) grown M and CCUG^rfp^ (or DE4373 or DE4381) strain cells ranged between 0.60 and 0.74 (data not shown).

In conclusion, the σ-factor mRNA levels varied in all four *M*. *marinum* strains dependent upon growth phase. The *sigB* and *sigE* transcripts dominated in stationary *M*. *marinum* cells irrespective of strain, suggesting that these two σ-factors play important roles for growth under these conditions. For others such as *sigC* and *sigD* the levels were higher in exponentially growing cells. There were also strain variations as exemplified by *sigM* and *sig3687* (Fig 3 and S4 Fig). The small difference in σ-factor mRNA levels in the M-strain data (exponential *vs*. stationary phase cells; S3 Fig panels F and G, and S4 Fig) might be due to the way this strain was cultivated [23].

### Levels of σ-factor mRNAs as a function of exposure to various stress conditions

*M*. *marinum* like other bacteria have developed sophisticated ways to adapt to different environmental stresses and the σ-factors play essential roles as switches that alter global gene expression patterns. This is also true when *M*. *marinum* infects a host (see below). Hence we decided to analyze the σ-factor mRNA levels in cells after exposure to different stress conditions (see Materials and Methods; [21]). These were: i) nitric oxide, ii) mitomycin C, iii) the antibiotic isoniazid, iv) osmotic stress, v) oxidative stress, vi) acidic stress, vii) starvation, viii) heat stress, ix) cold stress, and x) microaerobic stress (depletion of oxygen). Most of these conditions bear relevance to mycobacterial infections. The number of live cells after exposure to the different stress conditions was above 93% except for those cells exposed to heat stress. In this condition only 47±10% viable cells were detected after 24 h (S5 Fig). In the microaerobic conditions we monitored the σ-factor mRNA levels up to 12 days and the cells stopped growing within 3 days (see Materials and Methods and below). We conclude that the RNA used for RNASeq and qRT-PCR was extracted mainly from living cells but, for the heat-stressed cells we cannot exclude the possibility that the larger proportion of dead cells affected the quality of the RNA (but see also below).

**Fig 4:**
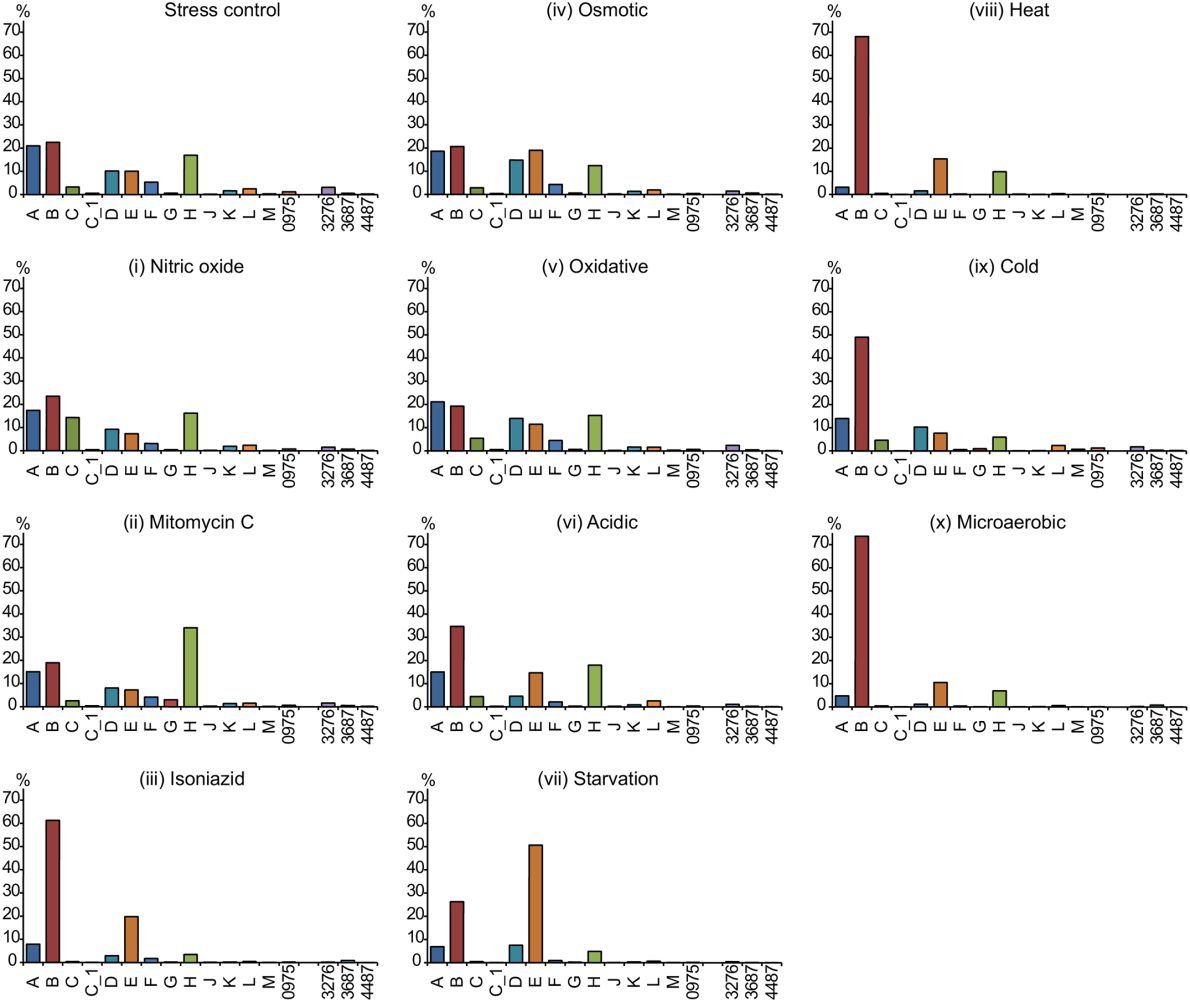
Distribution of σ-factor mRNAs in *M*. *marinum* CCUG^rfp^ exposed to various stresses. Cells were grown to exponential phase in liquid Middlebrook 7H9 medium followed by exposure to different stressors for 24 hours as indicated (except for microaerobic stress which lasted for 12 days; see Materials and Methods for details). The distribution (from RNASeq data) was calculated and plotted as described in figure legend 2. The roman numerals refer to the different stress conditions; for details, see main text.

**Fig 5:**
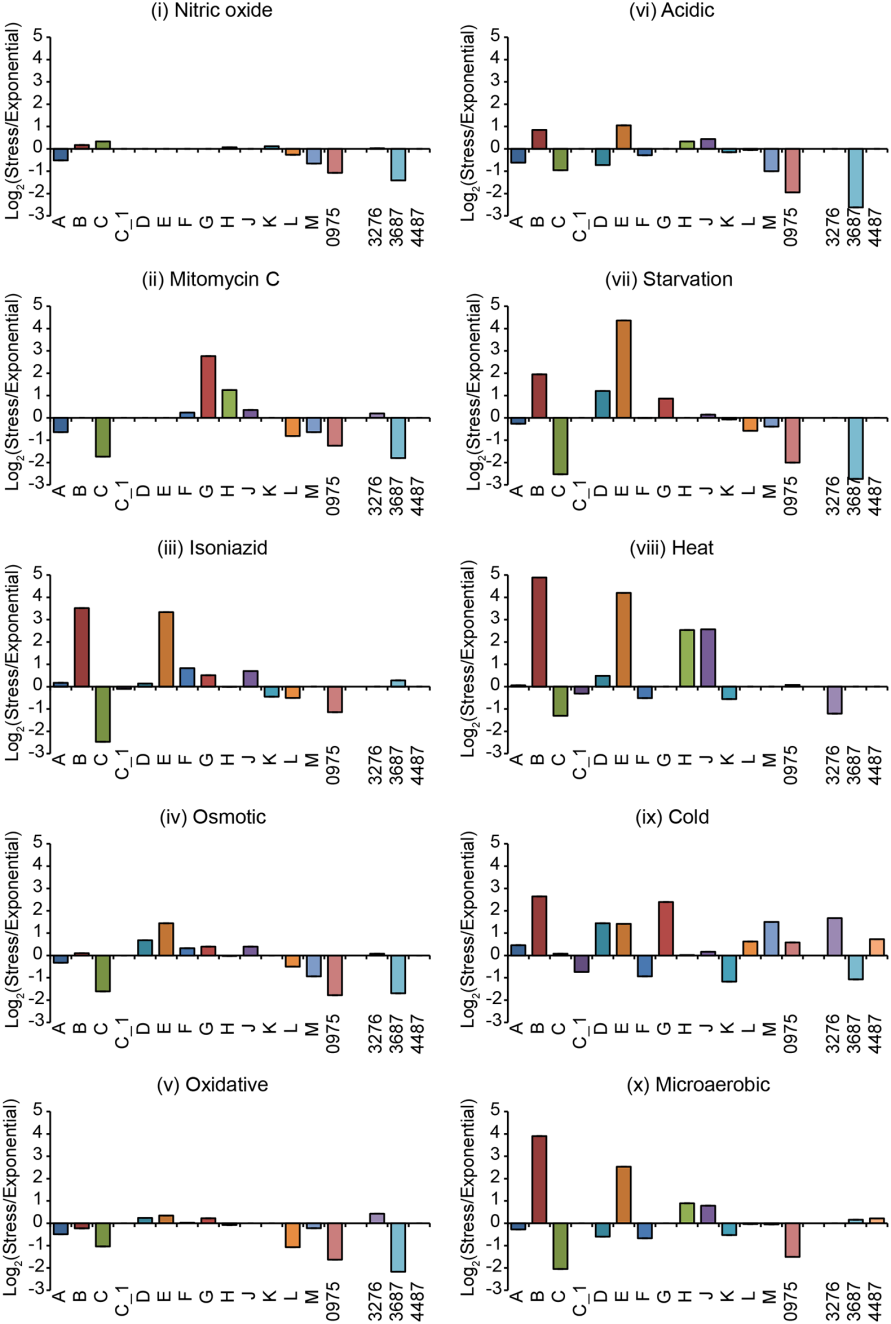
Change of σ-factor mRNA levels in response to different stress conditions. The σ-factor mRNA ratios from RNASeq data, calculated as GFOLD change values. A single replicate of stressed *vs* exponentially grown *M*. *marinum* CCUG^rfp^ cells after 24 hours of stress (12 days for microaerobic stress; see Materials and Methods for details) was plotted on a log_2_ scale. Positive values indicate higher mRNA levels after exposure to the stress conditions. The different σ-factors are marked on the x-axis. The roman numerals refer to the different stresses; for details, see main text.

**Fig 6:**
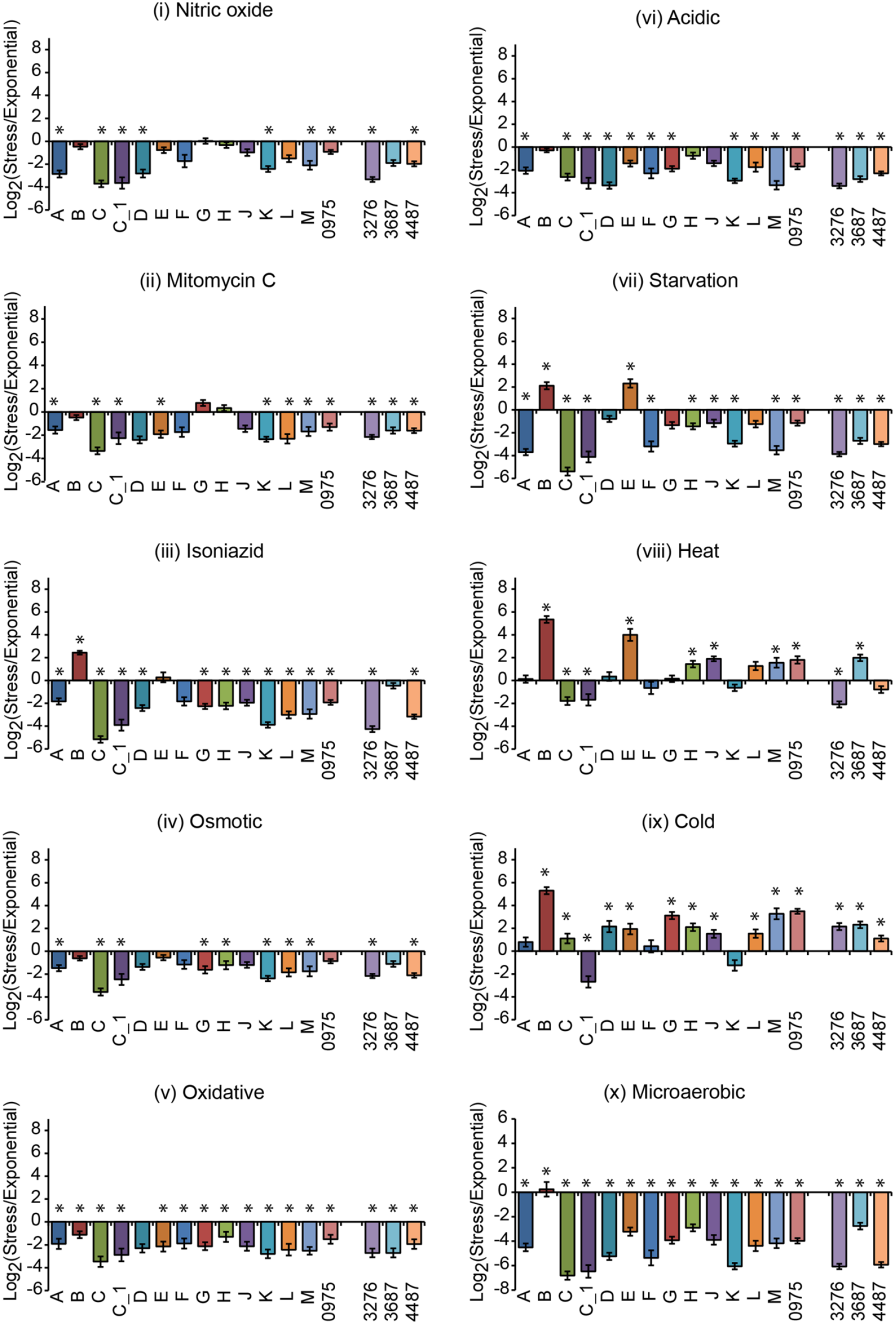
Change of σ-factor mRNA levels in response to different stress conditions. The σ-factor mRNA ratios, calculated from qRT-PCR data, of stressed and exponential (pre-stress control) cultures of *M*. *marinum* CCUG^rfp^ after 24 hours of stress (12 days for microaerobic stress; see Materials and Methods for details) plotted on a log_2_ scale. Positive values indicate higher mRNA levels after exposure to the stress. The different σ-factors are marked on the x-axis. The roman numerals refer to the different stresses, while stars indicate statistical significance, p < 0.05; for details, see main text. Significant log_2_-fold changes in mRNA levels for *sigB* 1.2 (iii), 1.1 (vii), 3.6 (viii), and 4.4 (ix) and for *sigE* 0.9 (vii), 2.6 (viii), and 0.9 (ix). For *sigH* and *sigJ* mRNAs (viii) log_2_-fold changes < 0.1 and 0.6, respectively. For *sig3687* mRNA the log_2_-fold changes were 0.7 (viii) and 1.2 (ix).

RNASeq was run on RNA extracted from cells exposed to the ten stressors/ stresses: (i) to (x). The data were analyzed for the distribution (Fig 4) and changes in σ-factor mRNA levels relative to the levels in exponentially growing cells. When we analyzed the changes in σ-factor mRNA levels we used both the RNASeq (Fig 5) and qRT-PCR (Fig 6) data sets (see above).

The distribution of the σ-factor mRNA levels in the *M*. *marinum* CCUG^rfp^ strain varied depending on stress condition (Fig 4). Exposure to nitrous stress (i), mitomycin C (ii), osmotic stress (iv), oxidative stress (v) and acidic stress (vi) resulted in similar distribution patterns with few changes. An increase in individual σ-factor mRNAs was detected for: *sigC* [in (i) and possibly also in (v) and (vi)], *sigH* and *sigG* [in (ii)], *sigE* [in (iv) and possibly also in (vi)] and *sigB* [in (vi)]; see Fig 5. For the other stress conditions [isoniazid (iii), starvation (vii), heat stress (viii), cold stress (ix) and microaerobic stress (x)] the patterns differed significantly from those for the stress-free control as well as for exponentially growing cells (referred to as pre-stress control; Fig 2A). The abundance of the *sigB* and *sigE* transcripts increased when exposed to isoniazid (iii), starvation (vii) and heat stress (viii) whereas only the *sigB* transcript showed an increased level for cold stress (ix) and microaerobic stress (x). In particular, the fractions were reduced for the *sigA*, *sigC* [except for cold stress (ix)], *sigD* [except for cold stress (ix)], *sigF*, *sigH* and *sig3276* transcripts.

The change in σ-factor mRNA levels after exposure to the various stress conditions revealed that the levels for several of the low abundant σ-factor transcripts changed in addition to those that were highly expressed such as *sigB* and *sigE*. Comparing the results using the RNASeq (Fig 5) and qRT-PCR (Fig 6) data sets were consistent in many cases but we also identified differences. We will therefore emphasize those cases where the two data sets were in agreement.

When exposed to nitric oxide (i), mitomycin C (ii), osmotic stress (iv), oxidative stress (v) and acidic stress (vi) small changes in mRNA levels were detected in keeping with the distribution data discussed above. (Note that the single replicate of the stress-free control indicated that the mRNA levels for all σ-factors decreased approximately 2 to 4 log_2_-fold as a result of growth, and that all statistically significant changes were lower in the stress exposed samples consistent with growth dependent changes; see S4 Fig) However, for mitomycin C (ii) the data were consistent with that the *sigG* transcript level increased in addition to the observed increase in *sigH* mRNA (for the qRT-PCR data statistical significance was not reached; S1 Table). A comparison of the σ-factor mRNA levels in response to the other stress conditions—exposure to isoniazid (iii), starvation (vii), heat stress (viii) and cold stress (ix)—also revealed significant increases for *sigB* mRNA and *sigE* mRNA (for statistical significance see legend Fig 6; the increase in *sigE* mRNA was not statistically significant after isoniazid exposure for the qRT-PCR data; S1 Table). Compared to the exponential pre-stress control the mRNA levels increased for several additional σ-factors in response to heat stress (viii) and cold stress (ix). This was most apparent from the qRT-PCR data (Fig 6). (Taking into consideration the growth dependent decrease in the mRNA levels detected in the stress-free control discussed above, almost all σ-factor mRNA levels increased in response to heat and cold stress.) Moreover, for heat stress (viii) we noted that both the RNASeq and the qRT-PCR data sets indicated increased mRNA levels for *sigH* and *sigJ* (Figs 5 and 6, but the significant changes were small see legend Fig 6). The expression of *sig3687* also increased significantly (see S1 Table and legend Fig 6) in response to heat stress (viii) and cold stress (ix). However, note that this was only apparent using the qRT-PCR data set. The genes that require the σ-factor Sig3687 for expression are not known, but it is plausible that this σ-factor is required for the expression of proteins (or non-coding RNAs) that are involved in the adaptation to these stress conditions.

In response to microaerobic stress (x), both the RNASeq and qRT-PCR data sets revealed clear differences, except for *sigB*. The reason is unknown but could be because the RNASeq data were related to the total RNA transcripts whereas we used 16S rRNA for the qRT-PCR. Nevertheless, irrespective of data set the level of *sigB* mRNA increased in response to microaerobic stress. Although the increase was small it was statistically significant for the qRT-PCR data (log_2_-fold change < 0.1). For a complete list of the log_2_-fold changes for all σ-factors and stress conditions (i) to (x), see S1 Table.

### Levels of σ-factor mRNAs as a function of time after exposure to heat stress, cold stress and microaerobic conditions

The data above were collected after 24 hours exposure to the different stress conditions (12 days for microaerobic stress). We therefore decided to study σ-factor mRNA levels by qRT-PCR as a function of time of exposure to heat- (viii), cold- (ix) and microaerobic stress (x) (see Materials and Methods and S6 Fig; for a complete list of p-values for all three stresses, see S5 Table).

Consistent with the data discussed above heat stress resulted in significantly increased levels of *sigB* and *sigE* transcripts already after one hour and maximum levels were reached after four hours. Expression of *sigG*, *sigH*, *sigM*, *sigJ* and *sig0975* also increased over time although less compared to *sigB* and *sigE* mRNAs. However, only the increase in *sigM* and *sig0975* mRNA levels were statistically significant but not until after 24 hours. Lower levels were detected for the other σ-factors such as *sigC*, *sigC_1* and *sig3276*. However, for several of the σ-factors the levels decreased after just a few hours of heat stress and then it appeared that the levels reverted to initial levels or increased upon longer exposure as exemplified by *sigK* and *sigL*. No significant changes were detected for *sigA*, the housekeeping σ-factor.

Cold stress increased the levels for all σ-factor mRNAs after just one-hour exposure (except for the *sigK* and *sigC_1* transcripts, which were lower at all time points), but the initial increases were only significant for ten of the σ-factors. Again the most apparent change was detected for *sigB* mRNA that increased steadily over time. Some, such as *sigA*, *sigC* and *sigF*, increased initially, then decreased (the initial increase in *sigF* mRNA was not significant). The levels for others (*e*.*g*., *sigD*, *sigE*, *sigM* and *sig0975*) remained significantly high throughout the time course while others (*e*.*g*., *sigG*, *sigH* and *sigJ*) increased steadily as did the *sigB* mRNA, but statistical significance was not reached until after 12 hours exposure to cold stress (*sigG* and *sigH* mRNAs) or not at all as for *sigJ* mRNA.

Microaerobic stress resulted in an early significant decrease for all σ-factor mRNAs except for *sigB*, which was statistically unchanged at all time points, and *sig3687* where the decrease was delayed and became significant after 6 days of incubation.

In conclusion, these data are consistent with the results discussed above. Together it suggests that *M*. *marinum* senses and adjusts the expression of the various σ-factors early after exposure to heat, cold and microaerobic stress. Moreover, together these data further suggest that increased expression of *sigB* and *sigE* is important to adapt to various stress conditions in *M*. *marinum* while others such as *sigG* is needed in response to exposure to more specific conditions such as exposure to the DNA damaging agent mitomycin C.

### *In vivo* σ-factor mRNA levels: mosquito larvae and during infection

**Fig 7:**
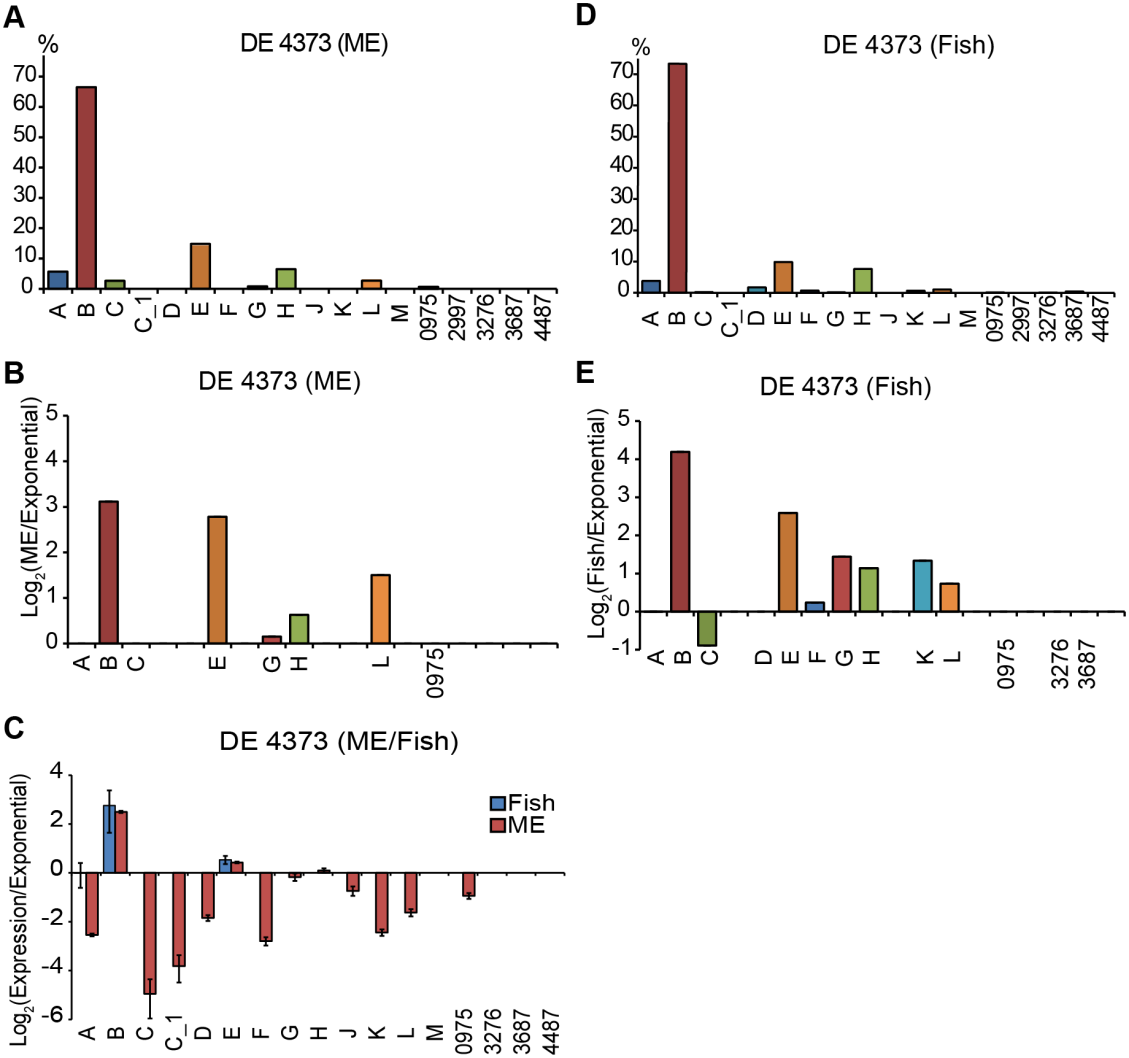
Distribution and change in *M*. *marinum* DE4373 σ-factor mRNA levels in fish and mosquito larvae. (A) and (D): The σ-factor mRNA distribution, calculated from RNASeq data and plotted as described in figure legend 2. (B), (C) and (E): Change in σ-factor mRNA levels in the DE4373 strain calculated from the RNASeq data (B and E), and qRT-PCR data (C) relative to the levels in exponentially growing (pre-stress control) *M*. *marinum* CCUG^rfp^ cultivated in 7H9 medium (see Materials and Methods for details). RNA extracted from DE4373 exposed mosquito larval (*Aedes aegypti*) (ME; A–C) and from pooled Japanese medaka livers (*Oryzias latipes*) (Fish; C–E). The mRNA ratios were plotted on a log_2_ scale. Positive values indicate higher mRNA levels when isolated from fish or ME. The different σ-factors are marked on the x-axis. Missing labels in (B) and (E) indicate that no reads were detected for those σ-factor mRNAs (in the fish or ME samples). Error bars in (C) indicate the variation within a triplicate assay of pooled biological replicates, while (A), (B), (D), and (E) represent data from one replicate. In (C), only *sigA*, *sigB*, and *sigE* could be reliably detected in DE4373 cells grown inside fish, while for the DE4373 RNA isolated from ME *sigM*, *sig3276*, *sig3687* and *sig4487* mRNAs could not be detected.

*M*. *marinum* can cause acute or chronic infections, depending on the bacterial dose, in fish such as the Japanese medaka (*Oryzias latipes*). It can also reside in aquatic invertebrate hosts like mosquito larvae or paramecium (*Paramecium caudatum*); the fish then becomes infected upon ingestion of the larvae. Interestingly, the passage of *M*. *marinum* through the gut of mosquito larvae increases virulence [14–16]. We therefore decided to study the *M*. *marinum* σ-factor mRNA levels by extracting RNA from *M*. *marinum* treated mosquito larvae and from infected Japanese medaka. In these experiments we used the *M*. *marinum* DE4373 strain (see Materials and Methods). The RNA was subjected to RNASeq and qRT-PCR. The data was processed as above. First we will discuss the "mosquito larvae" results and then the fish data (Fig 7).

#### Mosquito larvae

The distribution of σ-factor mRNAs revealed that *sigB* was the most abundant transcript followed by *sigE*, *sigH* and *sigA* (Fig 7A). This suggests that *M*. *marinum* was not growing exponentially in the larvae when the RNA was extracted (cf. Figs 2, 7A and S3 Fig; distribution of σ-factor mRNAs in exponentially growing *M*. *marinum* cells). Rather, the distribution of σ-factor mRNAs was more similar to that observed after exposure to heat (viii) and microaerobic stress (x) (Fig 4). When we compared the change of the different σ-factor mRNAs relative to the *in vitro* exponential pre-stress control *sigB* and *sigE* were increased as expected (Fig 7B). The RNA extracted from the DE4373 strain was also subjected to qRT-PCR and these data confirmed the increased levels of *sigB*, and possibly also *sigE* and *sigH* (although the increases were small) mRNAs, but not for *sigL* (Fig 7C). In addition the qRT-PCR data revealed small, if any, changes in the mRNA levels for *sigG*, *sigJ* and *sig0975* suggesting these σ-factors were expressed at similar levels as in exponentially growing cells.

#### Infected fish

Analysis of the RNA extracted from infected Japanese medaka by RNASeq revealed that the most abundant σ-factor transcript was *sigB* followed by *sigE*, *sigH* and *sigA* (Fig 7D). This distribution is similar to that observed after heat and microaerobic stress (Fig 4; note the CCUG^rfp^ strain was used in the heat and microaerobic stress experiments). These transcripts (except *sigA*, which was unchanged) and those from *sigG*, *sigK*, and *sigL* increased compared to the pre-stress control (Fig 7E). The increased levels of *sigB* and *sigE* mRNAs, as well as the unchanged *sigA* mRNA level, were confirmed by qRT-PCR (Fig 7C). The qRT-PCR experiment also revealed that the *sigA* mRNA was higher than in the stress-free control. We were unable to detect transcripts from the other σ-factor genes due to limiting amounts of RNA extracted from infected Japanese medaka.

Together these data suggests that *M*. *marinum* did not grow exponentially in the mosquito larvae or in the fish when the RNA was sampled. Rather, the σ-factor mRNA levels were more similar to those observed after exposure to heat or microaerobic stress.

### Levels of mRNAs of heat induced stress proteins Hsp, DnaK, DnaJ and ClpB

**Fig 8:**
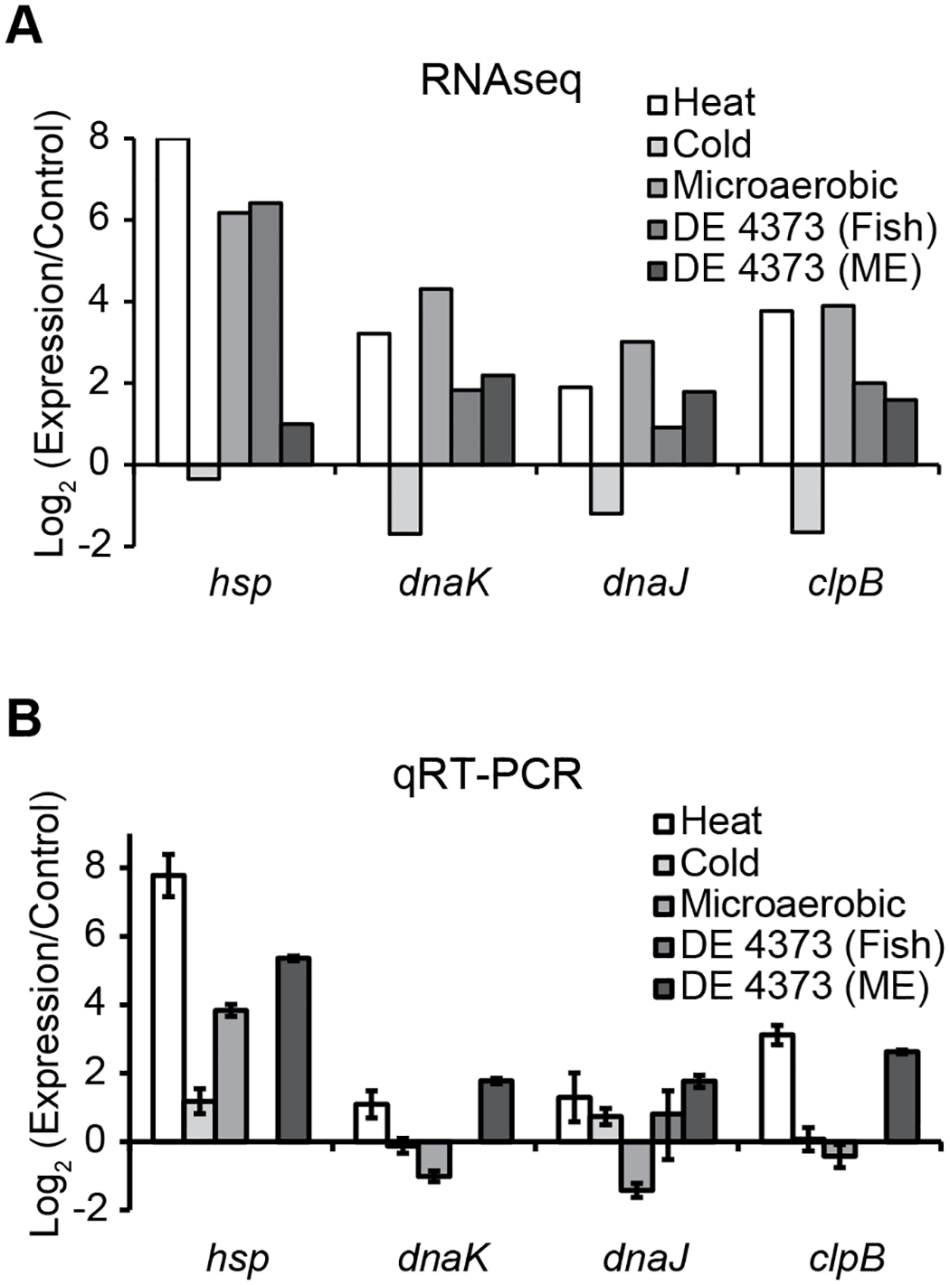
Change in the mRNA levels for selected heat shock induced genes. Change in the *hsp*, *dnaK*, *dnaJ*, and *clpB* mRNA levels: i) after exposure to heat, cold and microaerobic stress (in *M*. *marinum* CCUG^rfp^), ii) during infection of fish (*M*. *marinum* DE4373), and iii) in *M*. *marinum* DE4373 treated mosquito larvae (ME) relative to the levels in non-stressed (stress-free control) cells (see Materials and Methods for details). (A) Change in mRNA levels calculated from RNASeq data. The mRNA ratios are given as a generalized log_2_ fold (GFOLD) change of single replicates and plotted on a log_2_ scale such that positive values indicate higher mRNA levels after stress exposure, during infection, or in treated mosquito larvae. (B) Same as (A), but relative to the exponential pre-stress control. (C) Change in mRNA levels calculated from qRT-PCR data. Error bars indicate the variation within triplicate assays of one (Fish and ME; stress-free control) or two (heat, cold and microaerobic stress) biological replicates. In the fish, only *dnaJ* mRNA could be reliably detected by qRT-PCR.

To further understand whether the mRNA levels of heat and/ or microaerobic stress induced genes are also increased when *M*. *marinum* DE4373 reside in mosquito larvae or in infected fish we decided to monitor mRNA levels of the heat shock genes *hsp*, *dnaK*, *dnaJ* and *clpB*. In these experiments we only analyzed the change in mRNA levels relative to the stress-free control. The RNASeq data indicated that the levels for these genes increased upon in particular heat stress and under microaerobic conditions while a small decrease was detected after exposure to cold stress (Fig 8A and 8B; for the microaerobic, heat and cold stresses the CCUG^rfp^-strain was used). The increases were greater in the larvae than in the fish. We also monitored the levels by qRT-PCR (Fig 8C; in infected fish only *dnaJ* mRNA was detected). The qRT-PCR revealed higher transcript levels of *hsp*, *dnaK*, *dnaJ* and *clpB* after heat exposure and when *M*. *marinum* DE4373 reside in the mosquito larvae. Under microaerobic conditions only *hsp* mRNA was increased (Fig 8C). These findings are similar to the levels of the different σ-factor mRNAs in heat stressed *M*. *marinum* cells and when *M*. *marinum* habitat mosquito larvae. Together this might reflect that *M*. *marinum* is in a similar physiological state after exposure to heat stress and when residing inside mosquito larvae.

## Discussion

### Chromosomal organization of the mycobacterial sigma factor genes

The *M*. *marinum* CCUG strain carries 17 annotated σ-factor genes while other *M*. *marinum* strains such as the DE4373 and M strains have 18. The alternative σ-factor gene *sig2997* is missing in the CCUG-strain possibly due to a deletion as indicated from our genome sequencing analysis (Fig 1B). Our bioinformatics data suggested a chromosomal rearrangement that occurred after the divergence of *M*. *marinum* and *M*. *tuberculosis*. In particular, this rearrangement changed the position of *sigA*, *sigB*, *sigD*, *sigE*, *sigF*, *sigH* and *sigJ* (Fig 1A) relative to *dnaA* and *rpmH*, which both are located near *oriC* in many bacteria [24]. Rearrangement of σ-factor genes such as *sigA*, *sigB*, *sigD*, *sigF*, and *sigH* has also occurred during the evolution of *Mycobacterium ulcerans* Agy 99 (S1 Fig panel B), which conceivably diverged recently from *M*. *marinum*. These closely related *Mycobacterium* spp. might constitute ideal genomes to study the rules governing the evolution of bacterial genomes, as discussed by Röltgen et al. [25]. In this context it is interesting to note that *sigA*, *sigB*, *sigD*, *sigE*, *sigF*, *sigH* and *sigJ* are positioned roughly at the same locations relative to *dnaA* and *rpmH* as well as to other σ-factor genes in the more distantly related *M*. *marinum* and *M*. *smegmatis* mc^2^155 strains (Fig 1A).

### Sigma factor mRNA levels

All σ-factor genes in the different *M*. *marinum* strains studied in this report were expressed; however, the levels of expression of individual σ-factor genes varied dependent on growth conditions and exposure to different environmental stresses. This is in agreement with other reports on the expression of the 13 σ-factor genes in *M*. *tuberculosis* H37Rv grown under various conditions (for reviews see [5,8–10]). In addition, for *Mycobacterium avium* subsp. *paratuberculosis* (MAP) 19 putative σ-factor genes have been annotated and many of these were expressed under a variety of growth conditions including 7H9 media [26] (see also S1 Fig panel B).

A comparison of σ-factor gene transcript levels in exponentially growing *M*. *tuberculosis*, MAP and *M*. *marinum* cells in 7H9 liquid media suggests similarities as well as differences ([7,26]; this report). (Note that there is no growth phase information available for *M*. *avium* subsp. *paratuberculosis*; however, we use these numbers in our comparison). The *sigA*, *sigB*, *sigC*, *sigD*, *sigE* and *sigH* transcripts are among the most abundant in *M*. *tuberculosis* [7] and as shown here in *M*. *marinum* cells whereas it is *sigA*, *sigD*, *sigE*, *sigG* and *sigI* mRNAs that are most abundant in MAP cells [26]. Moreover, in exponentially growing *M*. *tuberculosis* cells the *sigC* transcript was the highest but not for *M*. *marinum* (and MAP), and especially not for the *M*. *marinum* M-strain [23] (Fig 2 and S3 Fig). Another difference is that the mRNA level of *sigH vs sigM* in *M*. *tuberculosis* is significantly lower compared to *M*. *marinum* [7] (Fig 2 and S3 Fig). In all four *M*. *marinum* strains, the levels of the *sigM* transcript were low in exponentially growing cells. It therefore seems that the expression profiles for the σ-factor genes in different *Mycobacterium* spp. vary in a species-specific manner in exponentially growing cells, even for closely related species such as *M*. *tuberculosis* and *M*. *marinum*. Conceivably this reflects that the expression of certain genes, which in many instances can be homologous genes, that are needed during exponential growth are regulated by different σ-factors in different *Mycobacterium*. It is worth noting that several of the σ-factor genes in *Mycobacterium* spp. are regulated by more than one promoter that in many cases overlap and these promoters are recognized by different σ-factors (see below).

### Sigma factor transcript profiles after exposure to stresses and inside infected fish or mosquito larvae

In *M*. *marinum* cells exposed to heat stress, microaerobic stress and during infection of Japanese medaka, the levels of individual σ-factor mRNAs were similar. The same is true for mRNA levels detected in *M*. *marinum* cells after passing through the mosquito larval digestive tract. In these four environments the most abundant mRNAs were those originating from *sigA*, *sigB*, *sigE* and *sigH*. In particular, high levels of *sigB* and *sigE* mRNAs were detected during infection. Exposure of *M*. *tuberculosis* cells to heat stress and low aeration also results in increased levels of *sigB*, *sigE* and *sigH* transcripts *in vitro* [7]. The σ-factor SigB is considered to be involved in the general stress response in *M*. *tuberculosis* and *M*. *smegmatis* and our data suggested that this is also the case for *M*. *marinum* [5,9,10,27] (see below). SigB is dispensable for *M*. *tuberculosis* growth *in vivo* [28] but it lowers the growth rate upon overexpression in macrophages [29]. The expression of the *M*. *tuberculosis sigE* and *sigH* genes also increases upon exposure to heat and during growth in mononuclear phagocytes. When deleted, effects on growth in macrophages and mortality in mice have been reported. These data demonstrate the importance of *sigE* and *sigH* during heat stress and indicate their role leading to a successful *M*. *tuberculosis* infection [7,30–34]. Together these data show similarities comparing the transcript levels of *sigB*, *sigE* and *sigH* in the phylogenetically closely related *M*. *marinum* and *M*. *tuberculosis* when they experience different growth environments. However, in the case of MAP no increase in *sigE* (as well as *sigB*) mRNAs was detected after 7 days of growth in intestinal epithelial cells [26] supporting the notion that the expression of individual σ-factors might vary in a species-specific manner (see above).

We also note that there seems to be a similarity with respect to the level of *sigG* mRNA, which increases both in *M*. *tuberculosis* and *M*. *marinum* upon exposure to the DNA cross-linking agent mitomycin C ([35]; for *M*. *marinum* the change in *sigG* was not statistically significant, see above and S1 Table). An increased *sigG* mRNA level is also detected when *M*. *tuberculosis* grows in human macrophages [36] and for *M*. *marinum* during infection and growth in fish (however, this was only observed using the RNASeq data set; Fig 7E). Noteworthy is that the role of SigG in the SOS response is controversial [37,38] and remains to be clarified; but see [39]. Moreover, we observed elevated levels of *sigJ* mRNA after heat stress, cold stress and in *M*. *marinum* cells grown for two weeks on solid 7H10 medium (but not in liquid cultures; Fig 3). The *sigJ* transcript level also increases in late stationary *M*. *tuberculosis* cells [40] and in MAP after either infection of Caco2 intestinal epithelial cells or growth in medium supplemented with mycobactin J and lysozyme (a medium that may mimic intracellular stresses; [26]). It therefore appears that there is also similarities with respect to *sigG* and *sigJ* mRNA levels when in particular *M*. *marinum* and *M*. *tuberculosis* (and conceivably also MAP) experience different growth environments. However, to establish this requires further experiments. Note also that the *M*. *tuberculosis* cells were much older than our *M*. *marinum* cultures and that the MAP cells were subjected to treatment resulting in a cell-wall deficient form [40,41].

Interestingly, comparing the levels of the housekeeping *sigA* mRNA shows an increase during *M*. *tuberculosis* growth in human macrophages and after 7 days of MAP infection of intestinal epithelial cells [26,42]. The enhanced level of *sigA* mRNA correlates with a higher intracellular growth rate for *M*. *tuberculosis* [42]. It is therefore conceivable that this is also the case for MAP. By contrast, for *M*. *marinum* we did not detect any change in the *sigA* mRNA level when grown in the fish (Fig 7C and 7E). Conceivably this might indicate a difference but to establish this warrants for further studies.

As mentioned above the mycobacterial σ-factor SigB is involved in the general stress response. This is consistent with our observation where we detected higher transcript levels not only in response to heat stress and microaerobic conditions but especially after exposure to cold stress, starvation and possibly also isoniazid (see S1 Table). The increased levels of *sigB* mRNA were accompanied with an increase in *sigE* mRNA under all these stresses except possibly for the isoniazid and microaerobic conditions (the RNASeq and qRT-PCR data sets were not consistent). In *M*. *tuberculosis* there are several promoters present upstream of *sigB* and its expression is regulated by SigB, SigE, SigF, SigH and SigL, and these play important roles for the expression of *sigB* under different growth conditions. Multiple promoters also exist for other σ-factor such as *sigE* (for reviews see [5,8–10]). The *sigB* promoter region in the *M*. *marinum* M- and CCUG-strains has a structure similar to that in *M*. *tuberculosis* (S7 Fig panel A). It is therefore likely that the regulation of the expression of *sigB* is also similar.

The effect of cold stress on the expression of genes in mycobacteria has not been extensively studied [7,43,44]. However, in *M*. *bovis* BCG *sigF* expression is induced [43] while in *M*. *tuberculosis* mild cold shock causes modest increases in the levels of *sigD* and *sigI* mRNAs ([7]; note that *sigI* is absent in *M*. *marinum*, see Fig 1). Our RNASeq and qRT-PCR data showed that the levels of most σ-factor mRNAs in *M*. *marinum* increased during cold shock. This suggests that many of these σ-factors are needed for the expression of downstream genes in response to cold. For example in *M*. *smegmatis* the level of the histone-like protein, Hlp, increases in response to cold shock [44]. Increase in the expression of histone-like proteins, nucleic acid-binding proteins and proteins involved in trehalose synthesis are reported for other bacteria. Trehalose is a sugar with a protective role for bacteria exposed to different stressors. In *E*. *coli*, the expression of some of the genes for trehalose synthesis depends on the σ-factor RpoS for expression [45–49]. This is consistent with the role of RpoS as a master regulator of the general stress response in *E*. *coli* [50]. For members of the *Mycobacterium* genus, additional studies and analysis of the transcriptome and proteome data will provide a comprehensive understanding of which proteins (and non-coding RNAs) that are up-regulated and needed in response to cold shock and other stress situations.

### Factors influencing expression of σ-factors and impact of transcription levels

The level of σ-factor mRNAs reflects transcription activities of the σ-factor genes, but these do not directly translate into changes in the levels or activities of the σ-factors themselves and consequently not to how much the changes in the levels of mRNA influence the expression of the genes under their control. To understand the expression and impact of σ-factors, we therefore also have to consider post-transcriptional regulation.

1. **mRNA stability.** Little is known about the RNA degradation and mRNA processing in *Mycobacterium*. The half-life of *sigA* mRNA in *M*. *tuberculosis* is >40 min while it is roughly 2 min for *sigB* mRNA [51]; this presumably affects the levels of the two proteins within the cell. The half-lives for the other σ-factor mRNAs have, to our knowledge, not been determined but most likely they vary.
2. **Promoter organization and translational start sites.** The expression level is also influenced by the efficiency and start of translation. For example, the translation of the *sigE* mRNA in *M*. *tuberculosis* can be initiated from three different start codons and the one selected depends on which promoter is used for generating the *sigE* mRNA [52]. Alignment of *sigE* from *M*. *marinum* (CCUG- and M-strains) and *M*. *tuberculosis* revealed similar gene structures (S7 Fig panel B). It is therefore plausible that expression of *sigE* and choice of translational start site in *M*. *marinum* mirror those reported for *M*. *tuberculosis*.
3. **Chromosomal location and organization of σ-factor genes.** A comparison of the location of the σ-factor genes on the *M*. *tuberculosis* and *M*. *marinum* chromosomes showed that several of the common σ-factor genes (*sigA*, *sigB*, *sigD*, *sigE*, *sigF*, *sigH* and *sigJ*) were positioned differently while the positions for the others (*sigC*, *sigG*, *sigK*, *sigL* and *sigM*) were roughly the same relative to *rpmH* and *dnaA* (and *oriC*; Fig 1A and S1 Fig). This suggests that a chromosomal rearrangement, conceivably a translocation, occurred after these two species diverged. Whether this chromosomal rearrangement has an impact on the expression of the σ-factor genes with changed positions remains to be investigated but, for example, the levels of *sigF* transcripts increase in stationary growing *M*. *tuberculosis* cells and upon exposure to oxidative stress [43,53]. This appeared not to be the case for *M*. *marinum* in which the level of *sigF* mRNA was higher in exponentially growing cell while there was no change after oxidative stress (this report; see also [21]). Chromosomal rearrangements may therefore have an impact on the expression of *sigF*.
4. **Impact of anti-σ-factors.** Another factor to consider is the presence of anti-σ-factors, *i*.*e*. proteins that bind to specific σ-factors and inhibit their activity. In *M*. *tuberculosis* anti-σ-factors have been identified for SigE, SigF, SigH, SigK and SigL [5] and orthologous genes encoding these anti-σ-factors are also present in *M*. *marinum*. Their levels, translation and interaction with the respective σ-factor will clearly affect σ-factor function and expression of the respective σ-factor regulons. Interestingly, we noted that for *sigF* the gene encoding the anti-SigF (*rsbW*, *usfX* in *M*. *tuberculosis*) is located upstream of *sigF* and that the putative translational stop codon for *rsbW* overlaps with the initiation codon for *sigF* both in *M*. *tuberculosis* and in *M*. *marinum*. This might indicate that expression of SigF depends on translational frame shifting (S7 Fig panel C). Moreover, our analysis of the *M*. *marinum* genomes predicted the presence of two putative anti-σ-factors that are not present in *M*. *tuberculosis*. One of them is encoded by MMAR2996 and it is located immediately downstream of the MMAR2997 σ-factor. Both these genes are missing in the CCUG-strain but present in the *M*. *marinum* M-strain in close vicinity of *sigC* (S1 Fig panel A). The putative anti-σ-factor gene, MMAR3277, is located downstream of *sig3276*. As for the other anti-σ-factor genes (except *rsbW*; see above) MMAR2996 and MMAR3277 are located immediately downstream of their respective σ-factor gene. Further experiments have to be performed to establish whether these function as anti-σ-factors. Preliminary data (to be published elsewhere) suggest that MMAR3277 is expressed. Finally, it is interesting to note that two-component signal systems influence the expression of specific σ-factors (see *e*.*g*., [52,54]) in *M*. *tuberculosis* and *M*. *smegmatis* whereas in other bacteria, such as *E*. *coli*, transcription repressors/ activators affect σ-factor expression [50].

### Concluding remarks

It is clear that the expression and regulation of the σ-factors in *Mycobacterium* depends on several factors and these questions need future attention. Our study provides a platform for understanding their expression and regulation in *M*. *marinum* that will be of importance to decipher the σ-factor regulons in *Mycobacterium*. In this context *M*. *marinum* is of particular interest because it exists in a variety of niches. These include natural aquatic environments (where it can form and grow in biofilms or as planktonic cells), survival and growth following ingestion by invertebrate vectors, and growth within target tissues inside infected fish. In these diverse environments, the different σ-factors play crucial roles by switching on different gene suites that prepare the bacteria for survival and/or growth. Finally, we want to emphasize the similarity of the σ-factor mRNA profiles for *M*. *marinum* in the mosquito larval gastrointestinal tract and that in infected fish tissues. It has been shown that there is a significant increase in virulence of *M*. *marinum* following passages through either the digestive tracts of mosquito larvae or digestive vacuoles of paramecia. We propose that this increase in virulence is due to induction of key gene suites that better prepare the bacteria to mount a successful infection [14–16].

## Materials and Methods

### Bacterial strains, media, and growth conditions

For the majority of the *in vitro* experiments, we used an *rfp*-tagged derivative of the *M*. *marinum* type strain CCUG T 20998 referred to as the CCUG^rfp^-strain (*M*. *marinum* CCUG T 20998 *attB*::*rfp-hygR*; smooth colony variant), while for the *in vivo* fish and mosquito larvae infection experiments, we used the *M*. *marinum* DE4373-strain, which is an *rfp*-tagged derivative of *M*. *marinum* 1218R (rough colony morphology; also a type strain, but obtained from another strain collection than CCUG T 20998; both CCUG T 20998 and 1218R are isolates of the ATCC 927 type strains). DE4373 has the *rfp* (red fluorescent protein) gene linked to the *kanR* gene integrated into the 1218R genome at the L5 *attB* site (*attB*::*rfp-kanR*). DE4373 was used since it was previously shown to efficiently infect fish [14]. *M*. *marinum* DE4381 is a derivative of *M*. *marinum* 1218S, which is a smooth colony variant of *M*. *marinum* 1218R ([22]; see [14,21] for details, see also S2 Table) and it was used for comparison. *M*. *marinum* CCUG T 20998 transformed with the pMS2Kan plasmid (kanamycin resistance, Kan^R^; [55]) was used for the "Live/ Dead" staining (LIVE/DEAD *Bac*Light Bacterial Viability Kit, Life Technologies) because the red fluorescence of the CCUG^rfp^-strain would likely interfere with the propidium iodide staining (also red fluorescence) of the "Live/ Dead" assay. Cells were grown in liquid 7H9 or on solid 7H10 media supplemented with appropriate antibiotics (100 μg/ml Hygromycin B or 25 μg/ml kanamycin), 10% oleic acid-albumin-dextrose complex (OADC), and 0.5% Tween–80 (only for 7H9 medium) according to the manufacturers recommendations. Cultures were incubated at 30°C (except where indicated) and the liquid cultures were shaken at 100 rpm.

### Stress induction

To minimize the risk of contamination of the stress cultures intended for stress sampling, a parallel culture was used for measuring optical density and to determine when to apply the stresses. To minimize the variation in inoculation density between this culture and the stress cultures, the start culture was diluted into a large master culture, which was then distributed into separate culture flasks. The exponential and stationary cultures, the stress-free control, and the four stress cultures–heat (42°C), cold (4°C), oxidative, and microaerobic—were prepared as described elsewhere [21]. Nitrous stress, isoniazid stress, and DNA damage stress were induced by adding appropriate volumes of a 1000x concentrated stock solution of DETA/NO (500 μM final concentration; [56,57]), isoniazid (25 μg/ml final concentration; at or just below the minimal inhibitory concentration, MIC, for isoniazid according to ([58,59]; our unpublished data suggested similar MIC values for the *M*. *marinum* CCUG strain), and Mitomycin C (0.5 μg/ml final concentration; [56]), respectively. Osmotic, acidic, and starvation stresses were induced by pelleting exponentially growing cells, then re-suspending them in the stress medium equilibrated at 30°C. For the osmotic stress, NaCl was added to the 7H9 medium to a final concentration of 500 mM [60]. The 7H9 medium in the acidic stress was acidified by addition of concentrated HCl to a final pH of 4.45 [60]. Exposure to starvation was performed by re-suspending the cells in phosphate buffered saline, PBS (10 mM Na_2_HPO_4_, 1.8 mM KH_2_PO_4_, 2.7 mM KCl and 137 mM NaCl, pH 7.4; [61]). After 24 hrs in the various stress conditions the cells were harvested by centrifugation at room temperature and flash-frozen in liquid nitrogen. To induce microaerobic stress, the cells were cultured without shaking and harvested after the disappearance of the blue color in a parallel culture treated with methylene blue, which monitors the presence of oxygen [56]. Cells were stored at -80°C until processed.

For the time series of heat, cold, and microaerobic stresses, duplicate cultures were grown in parallel. Immediately before stress induction in exponentially growing cells, and at various times thereafter, aliquots from the duplicate cultures were harvested as above. In the case of microaerobic stress, one culture was prepared for each time point and the disappearance of methylene blue was followed in parallel cultures.

### RNA extraction, RNA sequencing, cDNA conversion, and qRT-PCR analysis

RNA was extracted using Trizol in a bead beater followed by DNase treatment as previously described [21]. The resultant DNA-free RNA was then converted to cDNA as previously described [21], or submitted for RNA sequencing (RNASeq), which was performed at the SNP@SEQ Technology Platform at Uppsala University on a HiSeq2000 (Illumina) platform. Primers and probe design (listed in S3 Table), and conditions for quantitative real-time PCR (qRT-PCR) are described elsewhere [21]. Briefly, for relative quantification we used the standard curve method with 16S rRNA as the endogenous control and the stress-free control as the calibrator sample. TaqMan probes (S3 Table) were used and each replicate was assayed in triplicate on an ABI 7300 instrument. Each sample contained cDNA corresponding to 50 ng input RNA except for the RNA from the fish infected with DE4373 where the amount of starting material was limited.

### RNASeq data processing and analysis

#### Normalization of the RNASeq data

The RNA-sequencing generated 100 bps of paired-end reads with a total ranging between 2.7 to 9.1 million. Filtering of the low quality reads was performed using ConDeTri (version 2.2) [62], which trim and remove reads based on the quality score. Trimming of adaptor sequences and duplicate read filtering were done using Cutadapt (version 1.8) and FilterPCRduplicates (version 1.01), respectively [63]. The filtered, high quality reads for each sample were mapped to the reference genome of *M*. *marinum* M-strain using Bowtie2 [64]. Read counts for each of the samples were calculated and normalized by multiplying with size factors as described in DESeq normalization [65]. Normalized read counts were used to calculate gene expression values in RPKM (Reads Per Kilobase per Million). Percentage distribution of the σ-factor gene expression values (RPKM) was calculated by dividing each gene expression value with the sum of all the σ-factor expression values.

#### Differential gene expression analysis

Due to the lack of biological replicates in our RNASeq data, we calculated the differential gene expression in terms of the Generalized FOLD (GFOLD) change value using GFOLD (version 1.1.1) [66]. From the gene expression values, differentially expressed genes were identified in different samples and were represented as GFOLD values. GFOLD values can be considered as similar to the log_2_ fold change, which is presented in the figures for easier comparison. For the comparison between the different strains and growth phases, Pearson correlation R-values were calculated based on read counts.

### Statistical analysis

For the qRT-PCR data, where we at least had two biological replicates, the statistical significance of the changes was calculated. The qRT-PCR data was first log_2_ transformed to induce normality, and then the log_2_ values were tested using a two-tailed Student's t-test. The maximum hypothetical mean differences in log_2_-values between the respective samples that still resulted in statistical significance (p < 0.05) were also calculated (to one decimal's accuracy). These and/or the p-values are presented in S1 and S5 Tables and the significance is indicated in the respective figures.

For the mosquito extract and infected fish samples (both the RNASeq and the qRT-PCR data sets) the analyses were done on pooled biological replicates essentially representing a population average.

### Comparative σ-factor gene analysis

Protein sequences of the predicted CDS from all the listed *Mycobacterium spp*. [17,18,67,68] were aligned "all-versus-all" using BLASTp (version 2.2.30+) analysis [69] with minimum sequence identity of 45% and protein query coverage of at least 70%. BLAST output was parsed to identify orthologous genes between the *Mycobacterium spp*. using PanOCT (version 1.9) [70]. These data was used in the gene synteny figures.

### RNA extraction from mosquito larvae passed *M*. *marinum* and infected Japanese medaka

Individual fish (5 months old) were infected with *M*. *marinum* DE4373 as outlined below. After 6 weeks post infection, the fish were sacrificed and their kidneys and livers were recovered. The individual kidneys and livers were homogenized in 500 μL of PBS using a sterile pestle. To release the bacterial cells from the kidney-liver homogenate, 400 μL of this solution was mixed with 600 μL solution GP (50 mM Tris-HCl, 10 mM EDTA, 1% SDS, 30 mM sodium acetate), then transferred to a tube containing 0.5 g D matrix beads (MP Biomedicals, USA) and 650 μL acid phenol:chloroform (Ambion Life Technologies, USA). The tubes were bead-beated in two intervals of 40 sec each in the cold (with a 2 min pause in between). The bacterial portion was transferred to another tube containing B matrix beads (MP Biomedicals, USA) and processed in the bead beater in the cold for 45 seconds. The cells were centrifuged at 10000 rpm at +4°C for 10 min. The supernatant was transferred to a tube containing 500 μL 65°C acid phenol:chloroform, vortexed, incubated at 65°C for 10 min, and then vortexed again. The samples were centrifuged at 13000 rpm for 10 min and the supernatant was transferred to a fresh tube after which the RNA was extracted twice with 400 μL phenol:chloroform (1:1) and once with 400 μL chloroform. The RNA was precipitated by standard procedures and re-suspended in 30 μL RNase-free water. The RNA was DNase treated and RNA from 7 livers was pooled.

Isolation of RNA from *M*. *marinum* DE4373 cells that had passed through mosquito larvae (see above) was done in the same way except that only B matrix beads was used. Finally, RNA from several infected mosquito larvae was pooled.

The RNA was sequenced at the SNP@SEQ Technology Platform at Uppsala University on a HiSeq2000 (Illumina) platform or converted to cDNA for qRT-PCR analysis as previously described [21].

### Japanese medaka animal husbandry

All Japanese medaka propagations were performed essentially as described previously [12]. Briefly, fish from a transgenic line of Japanese medaka (*Oryzias latipes*) carrying multiple copies of a λ bacteriophage vector that was developed by Dr. Richard Winn (University of Georgia; [71]). They were propagated in the laboratory at the University of Louisiana with a maximum density of 30–40 adult per 10-gallon tank with a recirculating filtration and held at 28°C. All animal facilities were maintained with a photoperiod of 16 hours light and 8 hours dark cycle. Twice monthly, 20% of the water was changed. Fish were fed three times daily with combinations of Otohime, brine shrimp, or Aquatic Ecosystems high protein flakes as required.

For a standard infection experiment, 3–5 month old fish were transferred to a Biosafety Level 2 (BSL–2) laboratory at least two days prior to the *M*. *marinum* infection and were maintained in that facility for the entire duration of the experiment. A constant temperature of 28°C was maintained in the infection laboratory as well. Infected fish were either kept individually in the smaller 640 mL containers or in groups of ten in 3.7 liter aquariums. Water was changed weekly after transferring the fish to clean containers filled with carbon-filtered water pre-equilibrated at room temperature for at least 24 h.

### Mosquito larvae aquaculture

Yellow fever mosquito (*Aedes aegypti*) eggs were purchased from Benzon Research (Carlisle, Pennsylvania, USA) and hatched as per vendor's instructions. Environments with low oxygen levels facilitate and synchronize hatching of mosquito eggs, so they were cultured in aquarium water containing crushed fish flakes to boost bacterial growth and thereby diminish the dissolved oxygen levels. The water was taken from an occupied fish tank placed into a 1-liter beaker and incubated at 27°C for 24 to 48 hours. The eggs were submerged in the water for up to three days to allow maximal hatching time. Newly emerged mosquito larvae were transferred to 3.7 liter containers and raised for 7–11 days until they achieved maturation status. Larvae were housed in a BSL–2 laboratory (University of Lousiana, LaFayette, USA) for the duration of their development and fed once daily with crushed Aquatic Ecosystems high protein flakes.

### Infection of Japanese medaka with *M*. *marinum* in live mosquito larvae

Bacterial cultures (DE4373 strain) were grown individually in 5 mL broths to an OD_600_ of approximately 1.0 and adjusted to a final OD_600_ of 0.6 with PBS (see above). The 5 mL cultures were transferred to 640 mL containers and approximately 60–100 mosquito larvae were immersed into the bacterial suspensions. Larvae were exposed to the culture for 24 h prior to their use in experiments. Mosquito larvae were rinsed 4–5 times with deoxygenated aquarium water and fed to the fish over a one-week interval. Four meals of mosquito larvae (five larvae per meal) were given to each fish during this time period. Freshly prepared cultures and newly infected larvae were made separately for each meal. Mosquito larvae were inspected randomly under a fluorescent dissection microscope to confirm ingestion of the fluorescent bacteria. The larvae were washed up to four times in clean, filtered water, then, briefly put into ice water, to reduce their movement during microscopy. Larvae with high bacterial load (*i*.*e*., heavy fluorescence) were fed to the Japanese medaka.

### Quantification of *M*. *marinum* ingested by mosquito larvae

Bacteria ingested by mosquito larvae were quantified for each meal administered to the Japanese medaka. A sample of five larvae, equivalent to one meal, was visually inspected by microscopy to have ingested the appropriate strains of *rfp*-marked bacteria, as previously described. These larvae were then suspended in 500 μL PBS and homogenized with a small pestle in a microcentrifuge tube. Dilutions of the homogenate were plated in duplicate or triplicate on Middlebrook 7H10 plates containing 10% ADC, 0.5% glycerol, 100 μg/mL cycloheximide, 100 μg/mL ampicillin, and 20 μg/mL polymyxin B. Plates were incubated at 30°C for up to 10 days and colonies of fluorescent bacteria were counted. Average CFU (colony forming units) between two meals of larvae were recorded for each feeding (S4 Table).

### Ethics Statement

The fish handling procedures were conducted as prescribed by the National Institutes of Health-Biosafety in Microbiological and Biomedical Laboratories (NIH-BMBL) guidelines and were approved by the Institutional Biosafety Committee and the Institutional Animal Care and Use Committee at the University of Louisiana (Animal Assurance Identification number A3029-01 and IACUC approval No. 2014-8717-020). Specifically we abide by i) "The Guide for the care and use of laboratory animals, 8^th^ edition, 2010", ii) "The US PHS policy on humane care and use of laboratory animals" and iii) "The US Government principles for the utilization and care of vertebrate animals used in testing, research and training". The completed “ARRIVE guidelines checklist” is included in S1 File.

## Supporting Information

S1 Fig Organization of the σ-factor genes in different *Mycobacterium* species. The positions of the σ-factor genes on the respective chromosomes are shown in a circular head-to-tail alignment of the genomes for the (A) *M*. *marinum* CCUG, M, and 1218R; the latter is a variant of DE4373 (see Materials and Methods and S1 Table). (B) *M*. *marinum* CCUG, *M*. *ulcerans* Agy 99, and *M*. *avium* subsp. *paratuberculosis* MAP4. The *M*. *ulcerans* Agy 99, and *M*. *avium* subsp. *paratuberculosis* MAP4 σ-factor genes were downloaded from the NCBI database [70,71]. In (A), homologous σ-factor genes in the CCUG, M and 1218R strains are connected with green lines, while those present in the M and the 1218R (DE4373) genomes are connected with blue lines. In (B), homologous σ-factor genes in *M*. *marinum* CCUG, *M*. *ulcerans* Agy99 and *M*. *avium* subsp. *paratuberculosis* MAP4 are connected with green lines, while the homologous σ-factor genes present in *M*. *ulcerans* Agy99 and *M*. *avium* subsp. *paratuberculosis* MAP4 are connected with blue lines. The first and last genes of each genome, *dnaA* and *rpmH* are shown in bright red. (C), (D) and (E) show the local σ-factor gene synteny for the different *Mycobacterium* spp. Black arrows indicate the σ-factor gene, while light gray and dark gray arrows indicate homologous upstream and downstream genes. Open (white) arrows indicate non-homologous genes. Arrow labels refer to the respective gene or ORF name in the species or strain. Only σ-factor genes homologous to those annotated in *M*. *marinum* M are shown [17]. (C) σ-factor gene synteny for *M*. *marinum* CCUG, *M*. *marinum* M and *M*. *tuberculosis* H37Rv. Black arrows indicate the respective σ-factor gene, while light gray and dark gray arrows indicate homologous upstream and downstream genes. Open white arrows indicate non-homologous genes. Arrow labels refer to the gene or ORF name in the respective species or strain. Only σ-factor genes showing homology with those in *M*. *marinum* M are shown. For details see main text. In (D), *M*. *marinum* CCUG, *M*. *smegmatis* mc^2^155, and *M*. *avium* subsp. *paratuberculosis* MAP4 are compared, while (E) compares the *M*. *marinum* CCUG and 1218R (DE4373) strains with *M*. *ulcerans* Agy 99.
(PDF)

S2 Fig Growth curves of *M*. *marinum* CCUG^rfp^ in Middlebrook 7H9 medium. The growth curves are based on three independent experiments and the approximate sampling points of exponential (E), stationary (S), and non-stressed control (C) samples are marked. OD refers to optical density at 600 nm. For details see main text Materials and Methods.
(PDF)

S3 Fig Distribution of σ-factor mRNAs in different *M*. *marinum* strains. The data are plotted for *M*. *marinum* CCUG^rfp^ after one week of growth on solid Middlebrook 7H10 medium (A; CCUG^rfp^ 1w solid), and of *M*. *marinum* DE4373, DE4381, and M in early (Exponential; B, D, and F) and late (Stationary; C, E, and G) stages of growth in liquid Middlebrook 7H9 medium (see Materials and Methods). The distribution is given as the percentage of reads (from RNASeq data) originating from each individual σ-factor gene *vs*. the reads originating from all σ-factor genes. The different σ-factors are marked on the x-axis. For the M-strain, the calculations were based on data submitted to NCBI by [23] Wang et al. (2013); see main text.
(PDF)

S4 Fig Change of σ-factor mRNA levels in response to different growth states. (A) *M*. *marinum* CCUG^rfp^ grown for one week (1w) and two weeks (2w) on solid Middlebrook 7H10 medium (one replicate). (B) Exponential (Exp) and stationary (Stat) *M*. *marinum* DE4381 cells cultivated in liquid Middlebrook 7H9 medium (one replicate). (C) Exponentially (Exp) and stationary (Stat) *M*. *marinum* M cells. (D) *M*. *marinum* CCUG^rfp^ grown for one week (1w) (one replicate) and two days (2d) (two replicates) on solid Middlebrook 7H10 medium. (E) *M*. *marinum* CCUG^rfp^ grown for one week (1w) (one replicate) and two weeks (2w) (two replicates) on solid Middlebrook 7H10 medium. (F) Exponential pre-stress control (Exp) (at least 6 replicates) and stress-free control (Ctrl) (one replicate). *M*. *marinum* CCUG^rfp^ cells cultivated in liquid Middlebrook 7H9 medium. In (A)–(C) RNASeq data was used, while in (D)–(F) we used the qRT-PCR data. The GFOLD change values and the log_2_-fold changes (see Materials and Methods) were plotted on a log_2_ scale. Positive values indicate higher mRNA levels in later stages of growth. The different σ-factors are marked on the x-axis. For the M-strain, the calculations were based on data submitted to NCBI by [23]; see main text.
(PDF)

S5 Fig Cell viability after exposure to different stress conditions. The live/ dead staining kit (LIVE/DEAD *Bac*Light Bacterial Viability Kit, Life Technologies) was used to stain *M*. *marinum* T CCUG 20998/ pMS2Kan cells after exposure to different stresses. Time of sampling was 24 hours for all stress conditions except microaerobic stress (12 days). The average percentages of cells stained as dead (red fluorescence) were calculated from at least three independent experiments and the error bars indicate the experimental variation. The roman numerals refer to the different stress conditions, for details see the main text Materials and Methods.
(PDF)

S6 Fig Change in σ-factor mRNA levels as a function of time after stress induction. The change in σ-factor mRNA levels for *M*. *marinum* CCUG^rfp^ at 1, 4, 6, 12, and 24 hours (heat and cold stress), and at 9 hours, 1, 3, 6, and 12 days (microaerobic stress) after stress induction was measured by qRT-PCR. The mRNA levels for the individual σ-factors (S6A, *sigA* to *sigH* and S6B, *sigJ* to *sigM*, *sig0975*, *sig3276*, *sig3687* and *sig4487*) were plotted in relation to the levels before stress induction (0 hours/days). The results shown are averages of two independent experiments with experimental errors as indicated. Red, blue, and green color indicates heat, cold, and microaerobic stress data, respectively. Stars mark the statistical significances as follows: * p < 0.05; ** p < 0.01; *** p < 0.001.
(PDF)

S7 Fig Comparison of the promoter regions of *sigB* and *sigE*, and the *rsbW* and *sigF* junction. Sequence alignments of the promoter regions of *sigB* (A) and *sigE* (B), and the junction between the *rsbW* and *sigF* coding sequences (C) for *M*. *marinum* M, and *M*. *marinum* CCUG strains, and *M*. *tuberculosis* H37Rv. Consensus sequences for the different σ-factor recognition motifs are indicated with background shading and different font colors. Letters above the indicated sequences specify the corresponding σ-factors. The translational start codons are also marked with black boxes. In (B), binding sites for MprA are underlined, and three previously identified [54] alternative transcription start sites (P1, P2, P3) are indicated. In (C), boxes indicate the overlapping translational stop codon for *rsbW* (grey box) and start codon for *sigF* (black box).
(PDF)

S1 File NC3Rs ARRIVE Guidelines Checklist Pettersson et al. (PDF)

S1 Table Significant (p < 0.05) log_2_-fold changes and their associated p-values relative to exponential phase. (PDF)

S2 Table *M*. *marinum* strains used in this study. (PDF)

S3 Table Primers and probes for qRT-PCR analysis. (PDF)

S4 Table Colony count from infected fish organs. (PDF)

S5 Table Calculated p-values for the indicated times after stress induction relative to before induction. (PDF)
