# Supplementary figures and images for: Comparative Sigma Factor-mRNA Levels in Mycobacterium marinum under Stress Conditions and during Host Infection
Source: PLoS One. 2015 Oct 7;10(10):e0139823. doi: 10.1371/journal.pone.0139823 (PMC4596819; doi:10.1371/journal.pone.0139823)

A

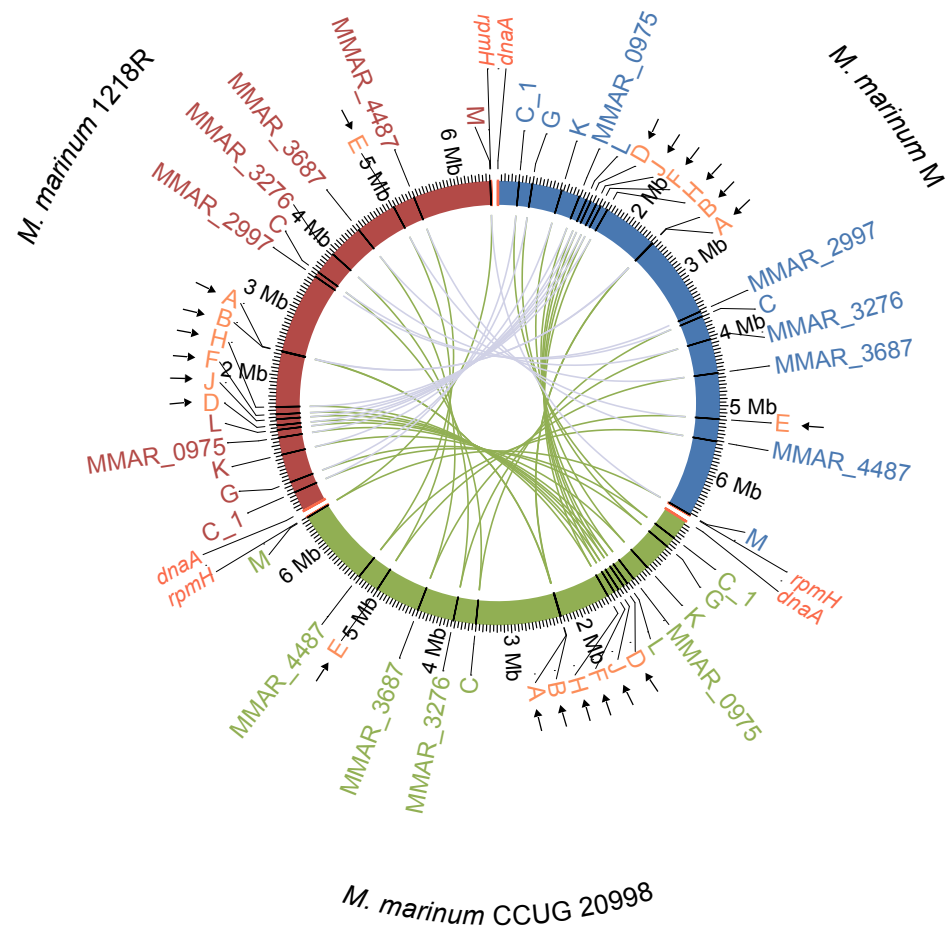

B

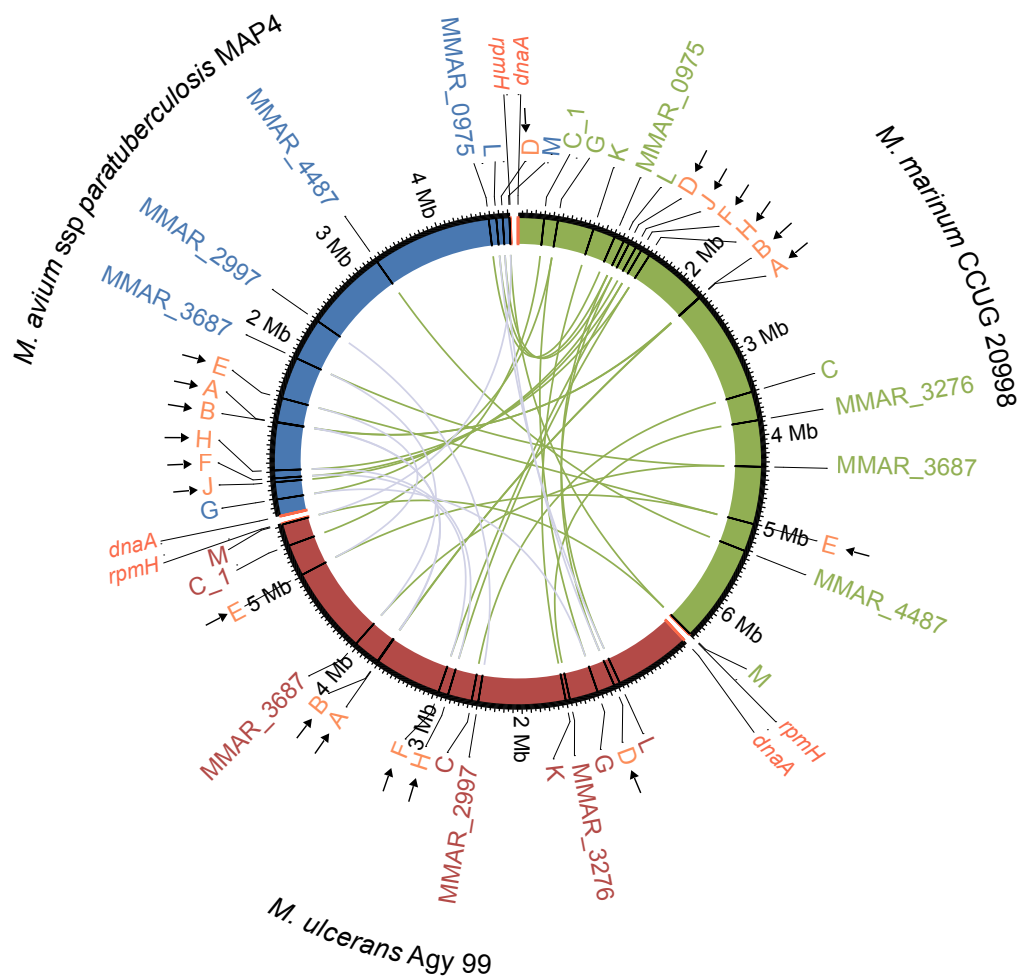

**C**

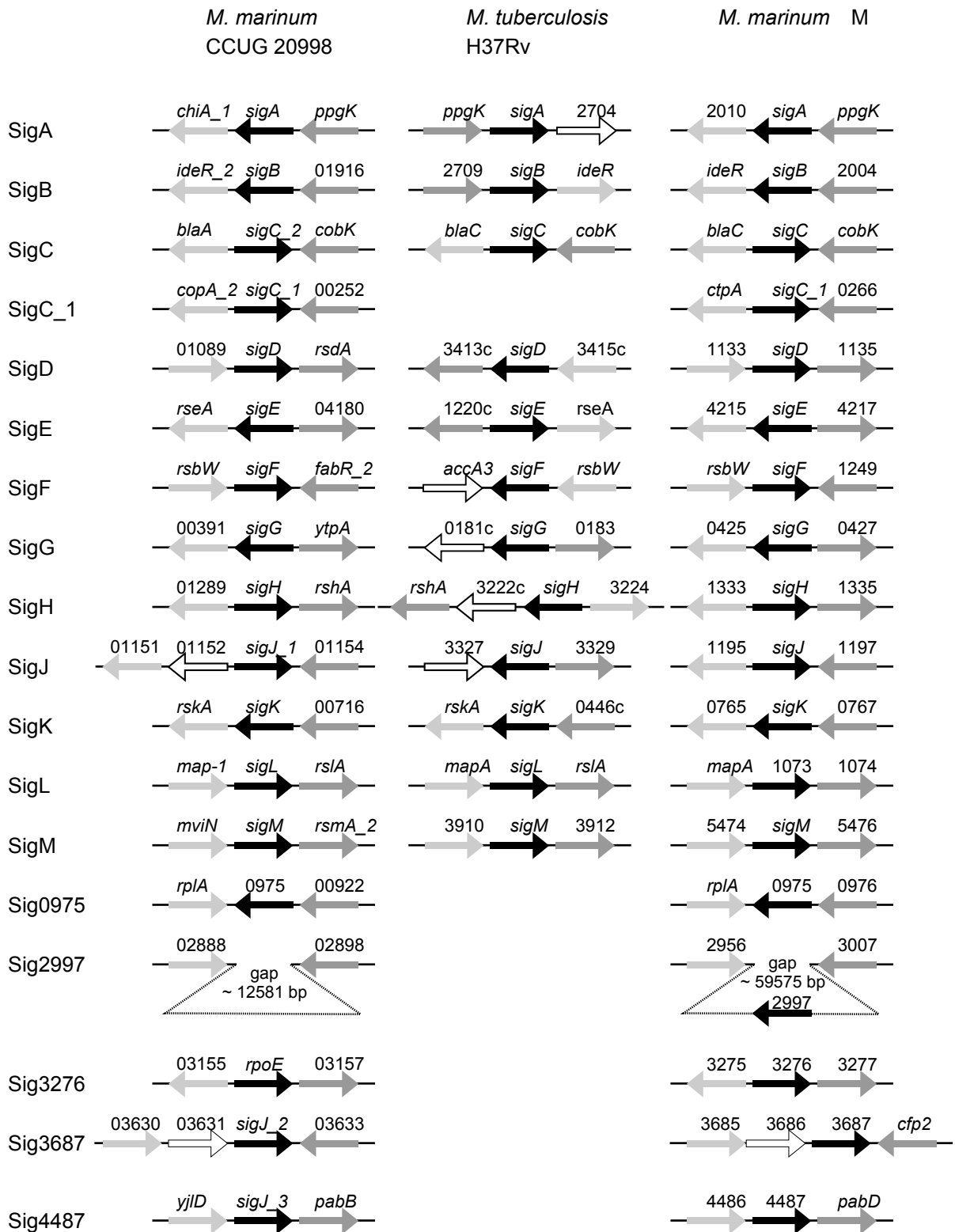

**D**

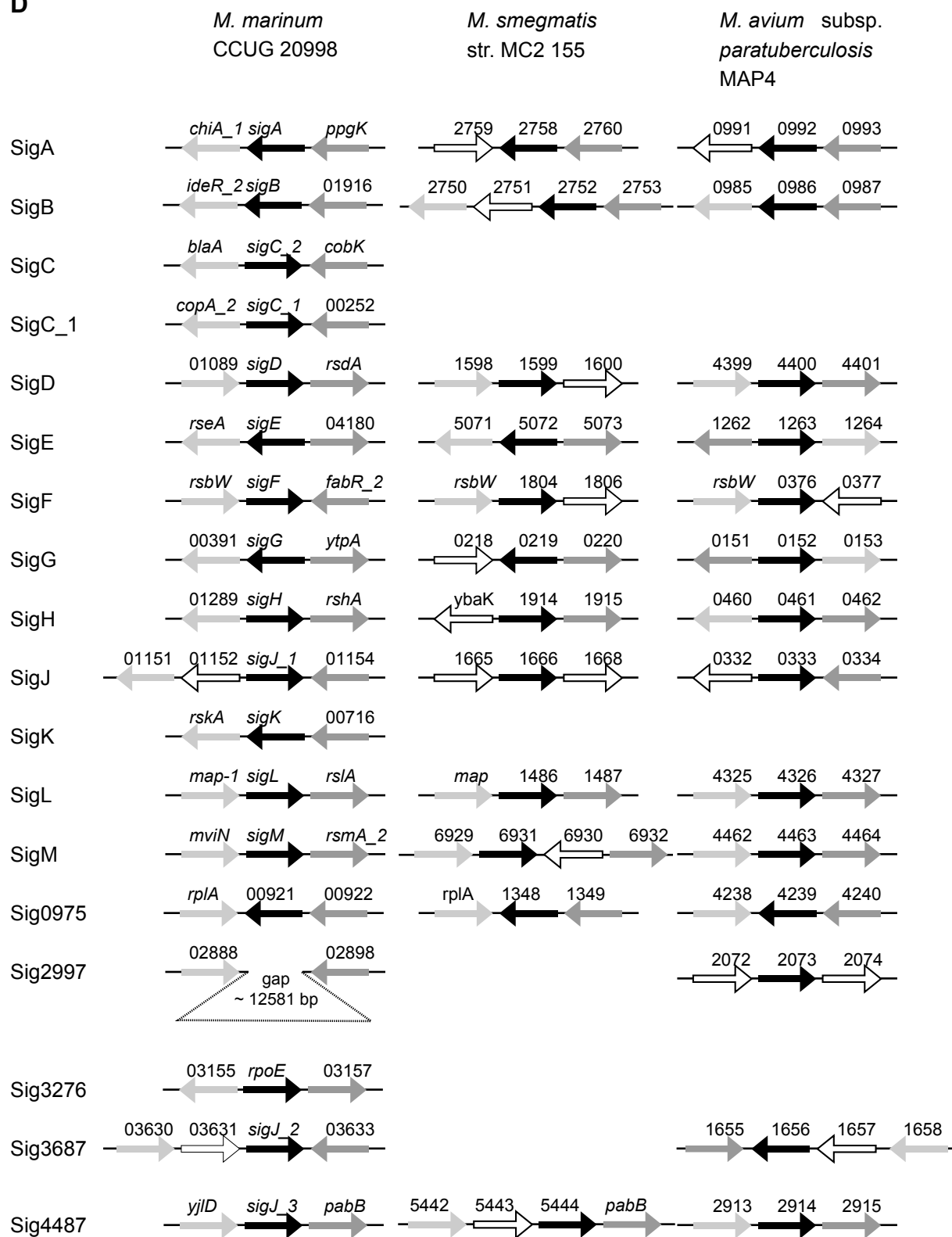

E

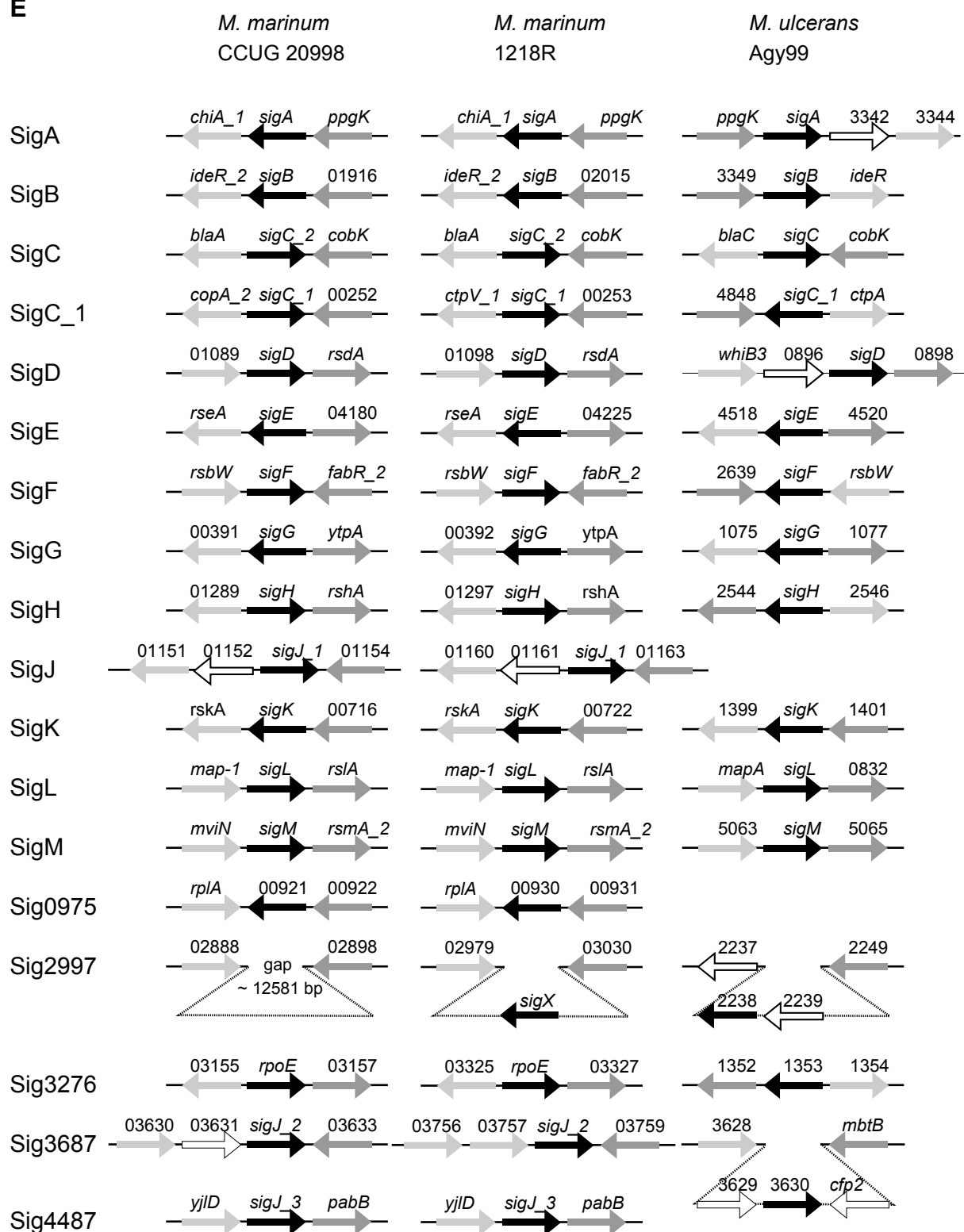

Supplement: S1 Fig — The positions of the σ-factor genes on the respective chromosomes are shown in a circular head-to-tail alignment of the genomes for the (A) M. marinum CCUG, M, and 1218R; the latter is a variant of DE4373 (see Materials and Methods and S1 Table). (B) M. marinum CCUG, M. ulcerans Agy 99, and M. avium subsp. paratuberculosis MAP4. The M. ulcerans Agy 99, and M. avium subsp. paratuberculosis MAP4 σ-factor genes were downloaded from the NCBI database [70,71]. In (A), homologous σ-factor genes in the CCUG, M and 1218R strains are connected with green lines, while those present in the M and the 1218R (DE4373) genomes are connected with blue lines. In (B), homologous σ-factor genes in M. marinum CCUG, M. ulcerans Agy99 and M. avium subsp. paratuberculosis MAP4 are connected with green lines, while the homologous σ-factor genes present in M. ulcerans Agy99 and M. avium subsp. paratuberculosis MAP4 are connected with blue lines. The first and last genes of each genome, dnaA and rpmH are shown in bright red. (C), (D) and (E) show the local σ-factor gene synteny for the different Mycobacterium spp. Black arrows indicate the σ-factor gene, while light gray and dark gray arrows indicate homologous upstream and downstream genes. Open (white) arrows indicate non-homologous genes. Arrow labels refer to the respective gene or ORF name in the species or strain. Only σ-factor genes homologous to those annotated in M. marinum M are shown [17]. (C) σ-factor gene synteny for M. marinum CCUG, M. marinum M and M. tuberculosis H37Rv. Black arrows indicate the respective σ-factor gene, while light gray and dark gray arrows indicate homologous upstream and downstream genes. Open white arrows indicate non-homologous genes. Arrow labels refer to the gene or ORF name in the respective species or strain. Only σ-factor genes showing homology with those in M. marinum M are shown. For details see main text. In (D), M. marinum CCUG, M. smegmatis mc2155, and M. avium subsp. paratuberculosis MAP4 are c [file pone.0139823.s001.pdf]

O. D. at 600 nm

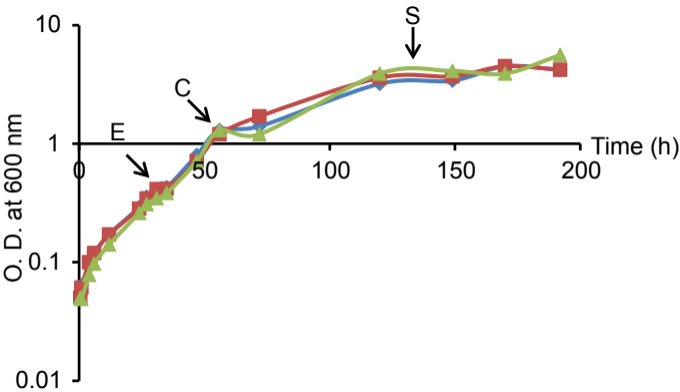

Supplement: S2 Fig — The growth curves are based on three independent experiments and the approximate sampling points of exponential (E), stationary (S), and non-stressed control (C) samples are marked. OD refers to optical density at 600 nm. For details see main text Materials and Methods. (PDF) [file pone.0139823.s002.pdf]

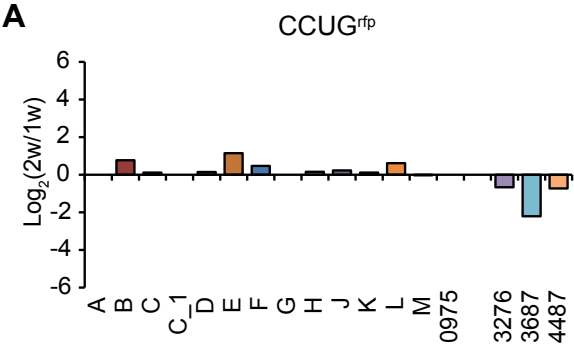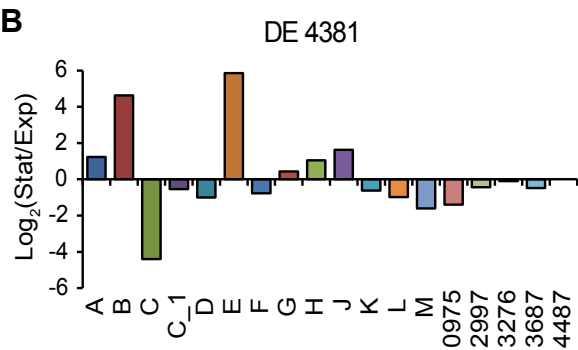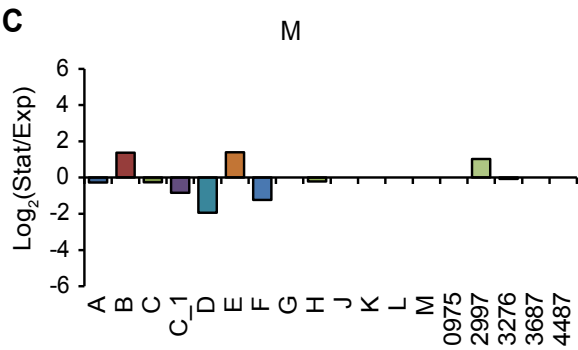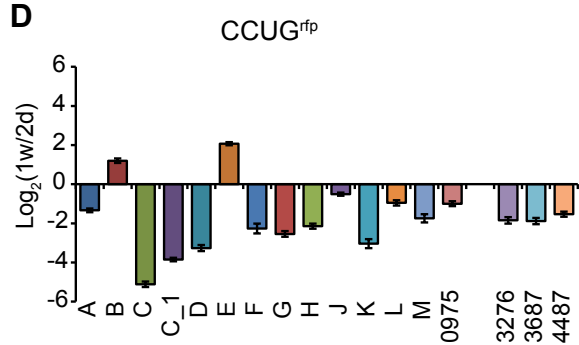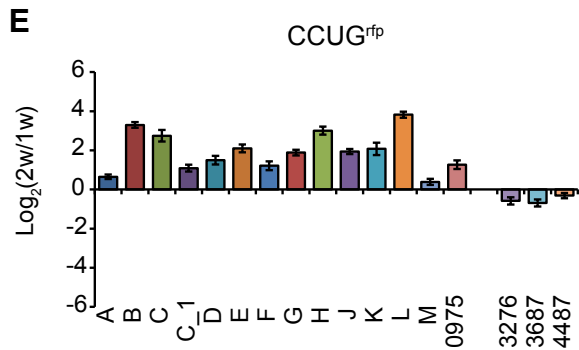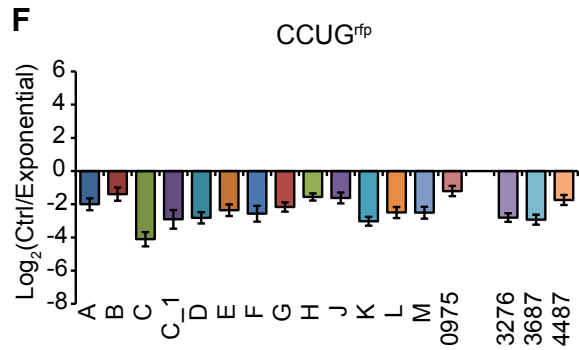

Supplement: S4 Fig — (A) M. marinum CCUGrfp grown for one week (1w) and two weeks (2w) on solid Middlebrook 7H10 medium (one replicate). (B) Exponential (Exp) and stationary (Stat) M. marinum DE4381 cells cultivated in liquid Middlebrook 7H9 medium (one replicate). (C) Exponentially (Exp) and stationary (Stat) M. marinum M cells. (D) M. marinum CCUGrfp grown for one week (1w) (one replicate) and two days (2d) (two replicates) on solid Middlebrook 7H10 medium. (E) M. marinum CCUGrfp grown for one week (1w) (one replicate) and two weeks (2w) (two replicates) on solid Middlebrook 7H10 medium. (F) Exponential pre-stress control (Exp) (at least 6 replicates) and stress-free control (Ctrl) (one replicate). M. marinum CCUGrfp cells cultivated in liquid Middlebrook 7H9 medium. In (A)–(C) RNASeq data was used, while in (D)–(F) we used the qRT-PCR data. The GFOLD change values and the log2-fold changes (see Materials and Methods) were plotted on a log2 scale. Positive values indicate higher mRNA levels in later stages of growth. The different σ-factors are marked on the x-axis. For the M-strain, the calculations were based on data submitted to NCBI by [23]; see main text. (PDF) [file pone.0139823.s004.pdf]

## Live/Dead staining

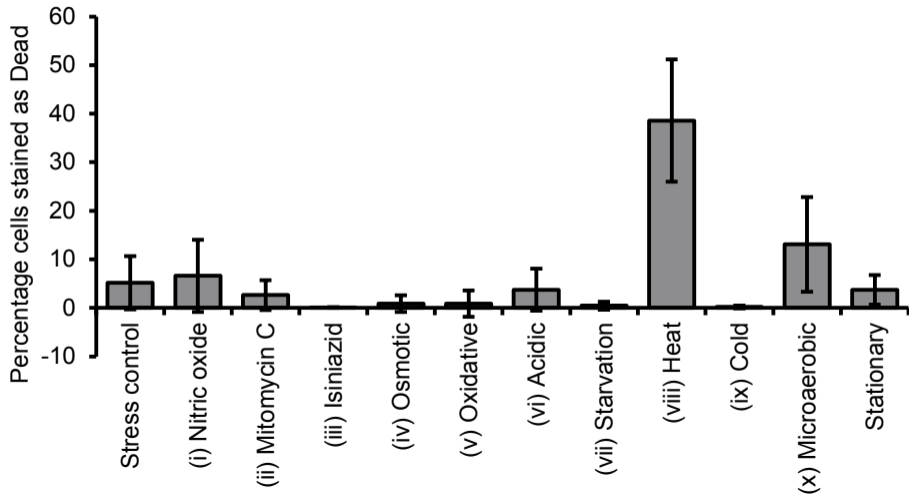

Supplement: S5 Fig — The live/ dead staining kit (LIVE/DEAD BacLight Bacterial Viability Kit, Life Technologies) was used to stain M. marinum T CCUG 20998/ pMS2Kan cells after exposure to different stresses. Time of sampling was 24 hours for all stress conditions except microaerobic stress (12 days). The average percentages of cells stained as dead (red fluorescence) were calculated from at least three independent experiments and the error bars indicate the experimental variation. The roman numerals refer to the different stress conditions, for details see the main text Materials and Methods. (PDF) [file pone.0139823.s005.pdf]

A

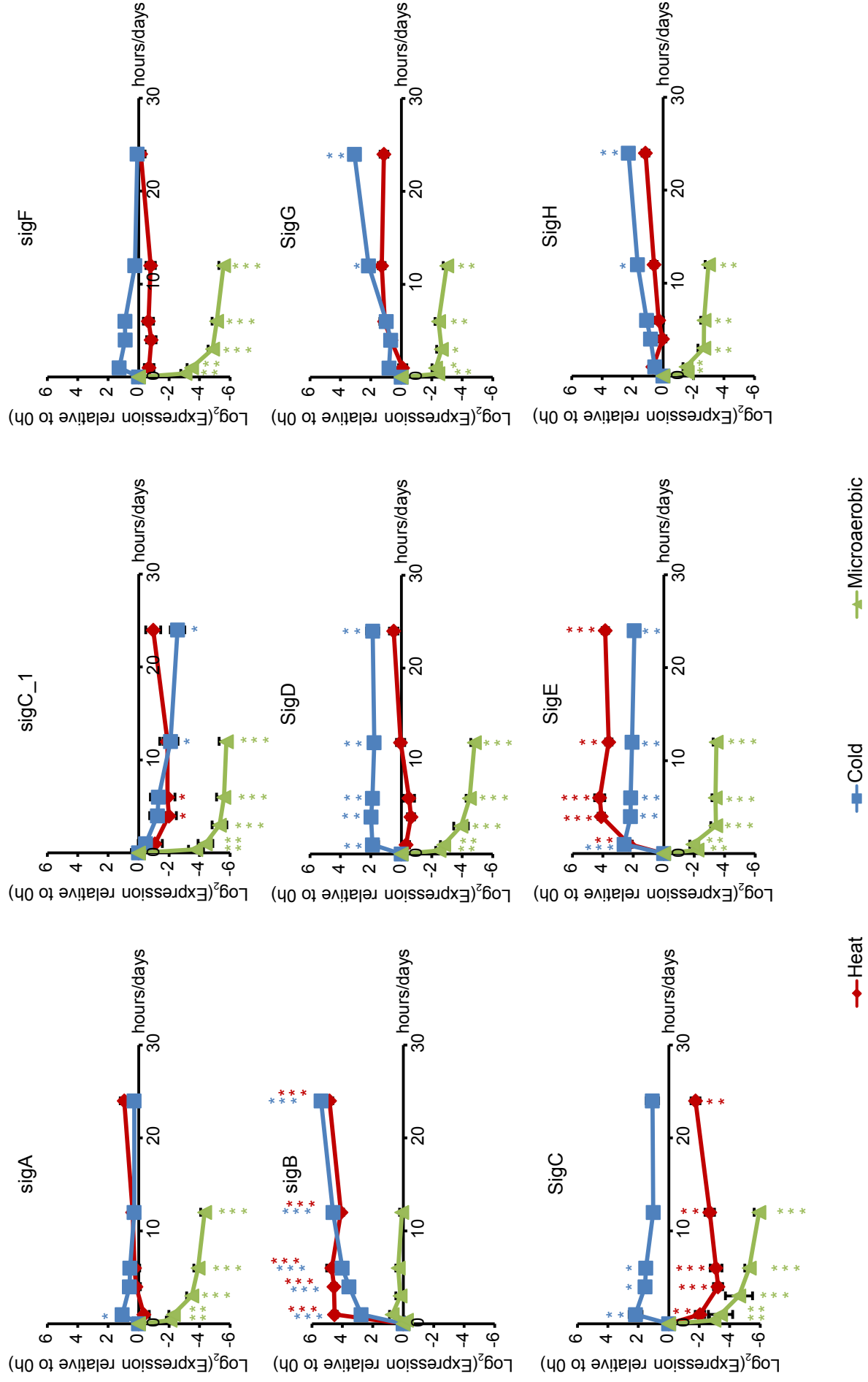

**B**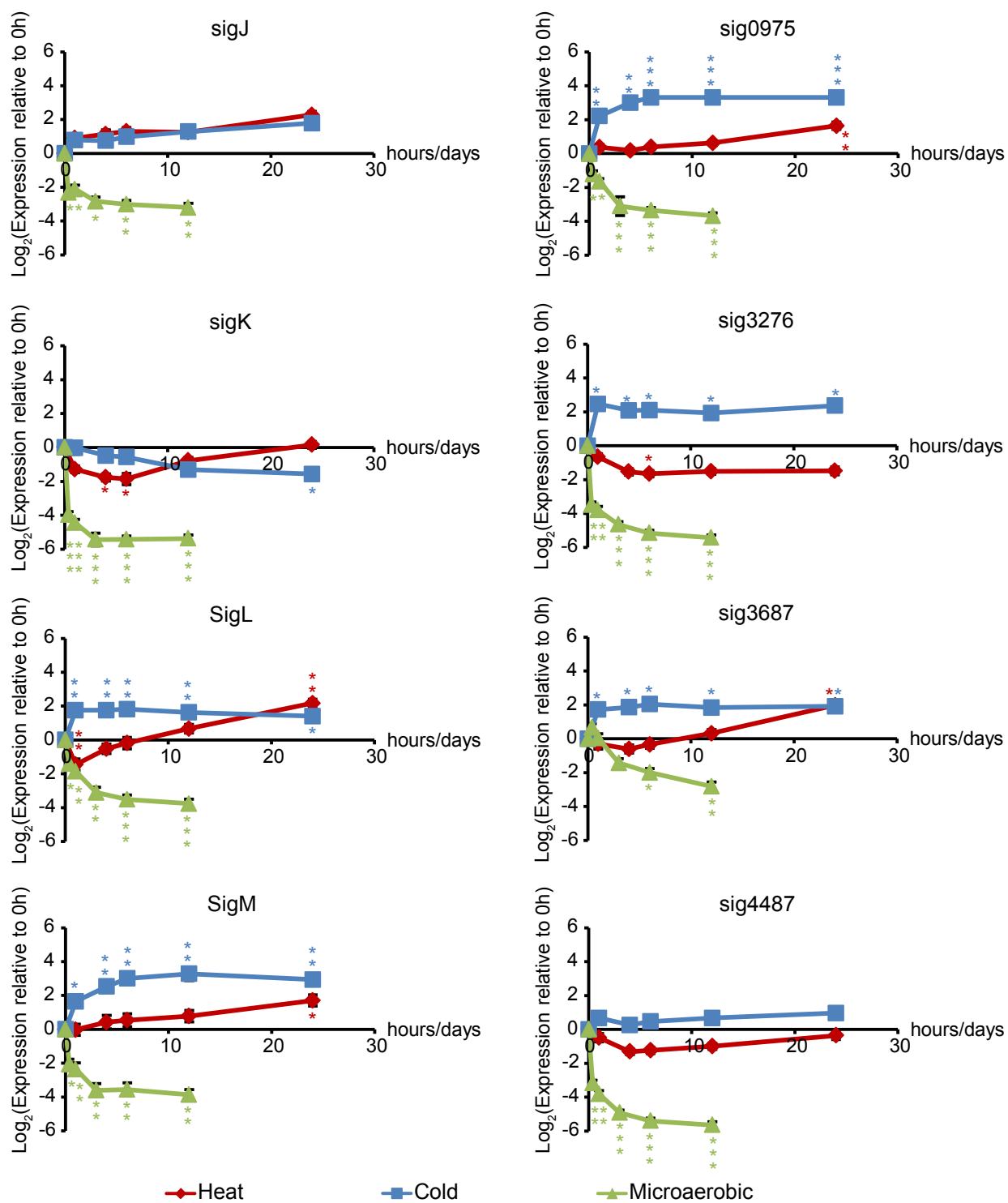

Supplement: S6 Fig — The change in σ-factor mRNA levels for M. marinum CCUGrfp at 1, 4, 6, 12, and 24 hours (heat and cold stress), and at 9 hours, 1, 3, 6, and 12 days (microaerobic stress) after stress induction was measured by qRT-PCR. The mRNA levels for the individual σ-factors (S6A, sigA to sigH and S6B, sigJ to sigM, sig0975, sig3276, sig3687 and sig4487) were plotted in relation to the levels before stress induction (0 hours/days). The results shown are averages of two independent experiments with experimental errors as indicated. Red, blue, and green color indicates heat, cold, and microaerobic stress data, respectively. Stars mark the statistical significances as follows: * p < 0.05; ** p < 0.01; *** p < 0.001. (PDF) [file pone.0139823.s006.pdf]
