# Supplementary material for: Comparative Sigma Factor-mRNA Levels in Mycobacterium marinum under Stress Conditions and during Host Infection
Source: PLoS One. 2015 Oct 7;10(10):e0139823. doi: 10.1371/journal.pone.0139823 (PMC4596819; doi:10.1371/journal.pone.0139823)
Supplement: S3 Fig — The data are plotted for M. marinum CCUGrfp after one week of growth on solid Middlebrook 7H10 medium (A; CCUGrfp 1w solid), and of M. marinum DE4373, DE4381, and M in early (Exponential; B, D, and F) and late (Stationary; C, E, and G) stages of growth in liquid Middlebrook 7H9 medium (see Materials and Methods). The distribution is given as the percentage of reads (from RNASeq data) originating from each individual σ-factor gene vs. the reads originating from all σ-factor genes. The different σ-factors are marked on the x-axis. For the M-strain, the calculations were based on data submitted to NCBI by [23] Wang et al. (2013); see main text. (PDF) [file pone.0139823.s003.pdf]

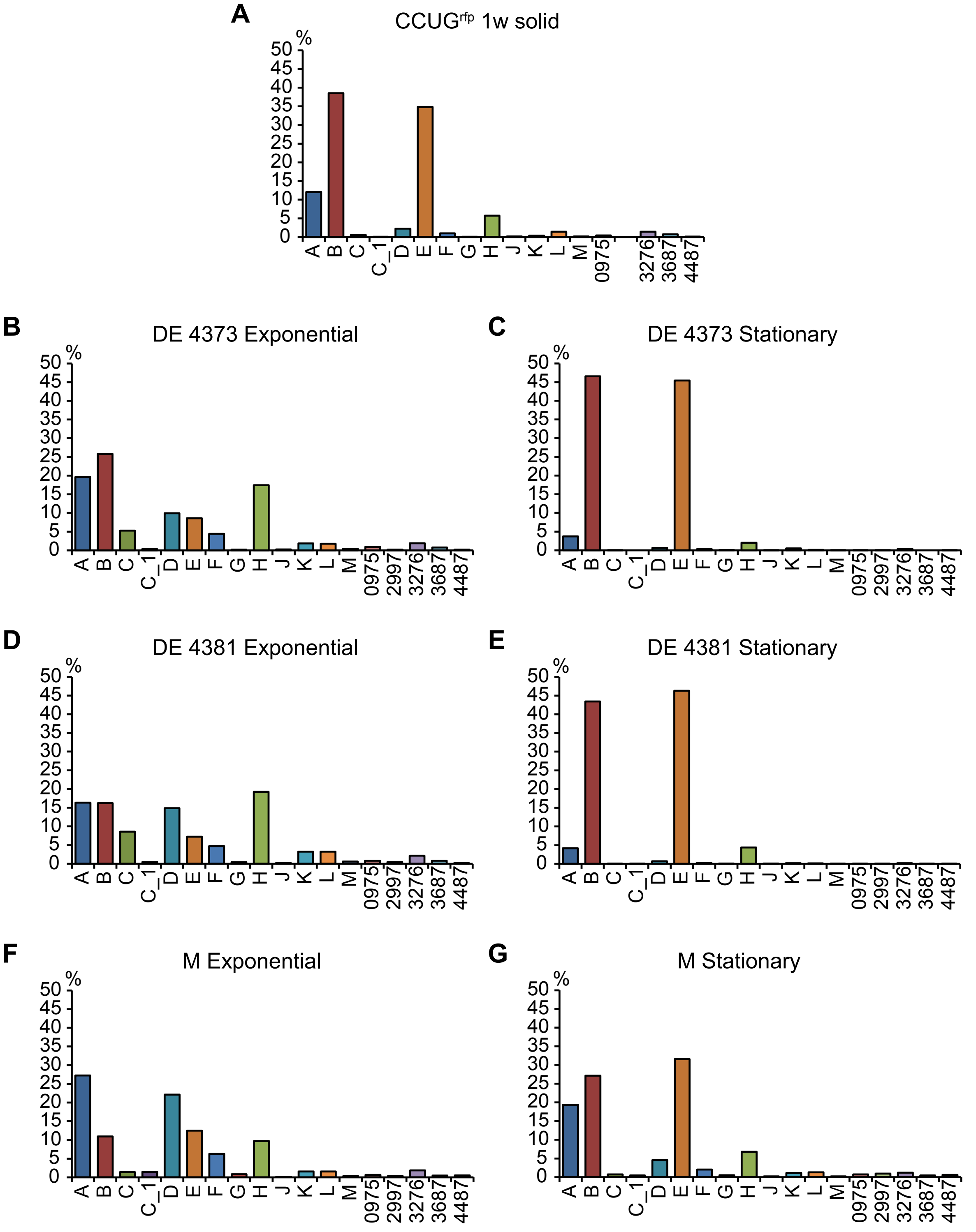
A
CCUG rfp 1w solid
B
DE 4373 Exponential
C
DE 4373 Stationary
D
DE 4381 Exponential
E
DE 4381 Stationary
F
M Exponential
G
M Stationary
%
50
45
40
35
30
25
20
15
10
5
0
A
B
C
C_1
D
E
F
G
H
J
K
L
M
0975
2997
3276
3687
4487
