# Supplementary material for: Comparative Sigma Factor-mRNA Levels in Mycobacterium marinum under Stress Conditions and during Host Infection
Source: PLoS One. 2015 Oct 7;10(10):e0139823. doi: 10.1371/journal.pone.0139823 (PMC4596819; doi:10.1371/journal.pone.0139823)
Supplement: S7 Fig — Sequence alignments of the promoter regions of sigB (A) and sigE (B), and the junction between the rsbW and sigF coding sequences (C) for M. marinum M, and M. marinum CCUG strains, and M. tuberculosis H37Rv. Consensus sequences for the different σ-factor recognition motifs are indicated with background shading and different font colors. Letters above the indicated sequences specify the corresponding σ-factors. The translational start codons are also marked with black boxes. In (B), binding sites for MprA are underlined, and three previously identified [54] alternative transcription start sites (P1, P2, P3) are indicated. In (C), boxes indicate the overlapping translational stop codon for rsbW (grey box) and start codon for sigF (black box). (PDF) [file pone.0139823.s007.pdf]

A

```
                                   10        20        30        40    E/H  E/H
M. marinum M            1 ACGACGCCCGGCGGCATACCCCCCTGTTCCCCACCGCTGAGCGGTTGGCGTTGGACGCCG 60
M. marinum CCUG 20998   1 ------------------------------CCACCGCTGAGCGGTTGGCGTTGGACGACG 30
M. tuberculosis H37Rv   1 -----------------------GAACGGCCCGCACTCGAGCCGC-GGCGACAGCCGGCA 36

                                   70        80        90   G    100       110
M. marinum M           61 GGCTGGAAGACCCCGCGCCACGGTGGGACGCGCAGCGCTTCGACGAGCGGG--GCTAGTC 118
M. marinum CCUG 20998  31 GGCTGGAAGACCCCGCGCCACGGTGGGACGCGCAGCGCTTCGACGAGCGGG--GCTAGTC 88
M. tuberculosis H37Rv  37 GAGCGGTCAGCCCCGCG-------GGGATTCGCCG-ACCACGGTTAGCCGTCTGTTGGCC 88

                             H     130 E/H   140       150       160       170
M. marinum M          119 CGGGTTCCGGTCAACCGACCCGGCGACACATTTCTCAGGACATTCTCAGCTCGGCGGCAC 178
M. marinum CCUG 20998  89 CGGGTTCCGGTCAACCGACCCGGCGACACATTTCTCAGGACATTCTCAGCTCGGCGGCAC 148
M. tuberculosis H37Rv  89 GGCGTTCCGGGTTGTCGGCCACTGGCCACACTTCTCAGGACTTTCTCAGGTCTTCGGCAG 148

                                   190 M     200 M     210       220       230
M. marinum M          179 ATTCCTGCACGTCACGGGGTGTGAGATGACAGCTGCGCGGGAACTCTGCGGTGGAACCTG 238
M. marinum CCUG 20998 149 ATTCCTGCACGTCACGGGGTGTGAGATGACAGCTGCGCGGGAACTCTGCGGTGGAACCTG 208
M. tuberculosis H37Rv 149 ATTCCTGCACGTCACAGGGCGTCAGATCACTGCTGGGTGGGAACTCAAAGTCCGGCTTTG 208

                            D      250       260       270       280       290
M. marinum M          239 TCGTTAATCCTCATGACAGCACAAGCCGATCGGGAGGGCGAAATGGCACAGCCCATAGCA 298
M. marinum CCUG 20998 209 TCGTTAATCCTCATGACAGCACAAGCCGATCGGGAGGGCGAAATGGCACAGCCCATAGCA 268
M. tuberculosis H37Rv 209 TCGTTAAACCCCATGACAGTGCAAGCCGATCGGGAGGTCGCTATGGCCGATGCACCCACA 268

                                   310       E/H       330
M. marinum M          299 AG-------------------------------                            300
M. marinum CCUG 20998 269 AGGGGCATCGTAGGCCGGGTTGACAGCGATCT-                            300
M. tuberculosis H37Rv 269 AGGGCCACCACAAGCCGGGTTGACAGCGATCTG                            301
```

B

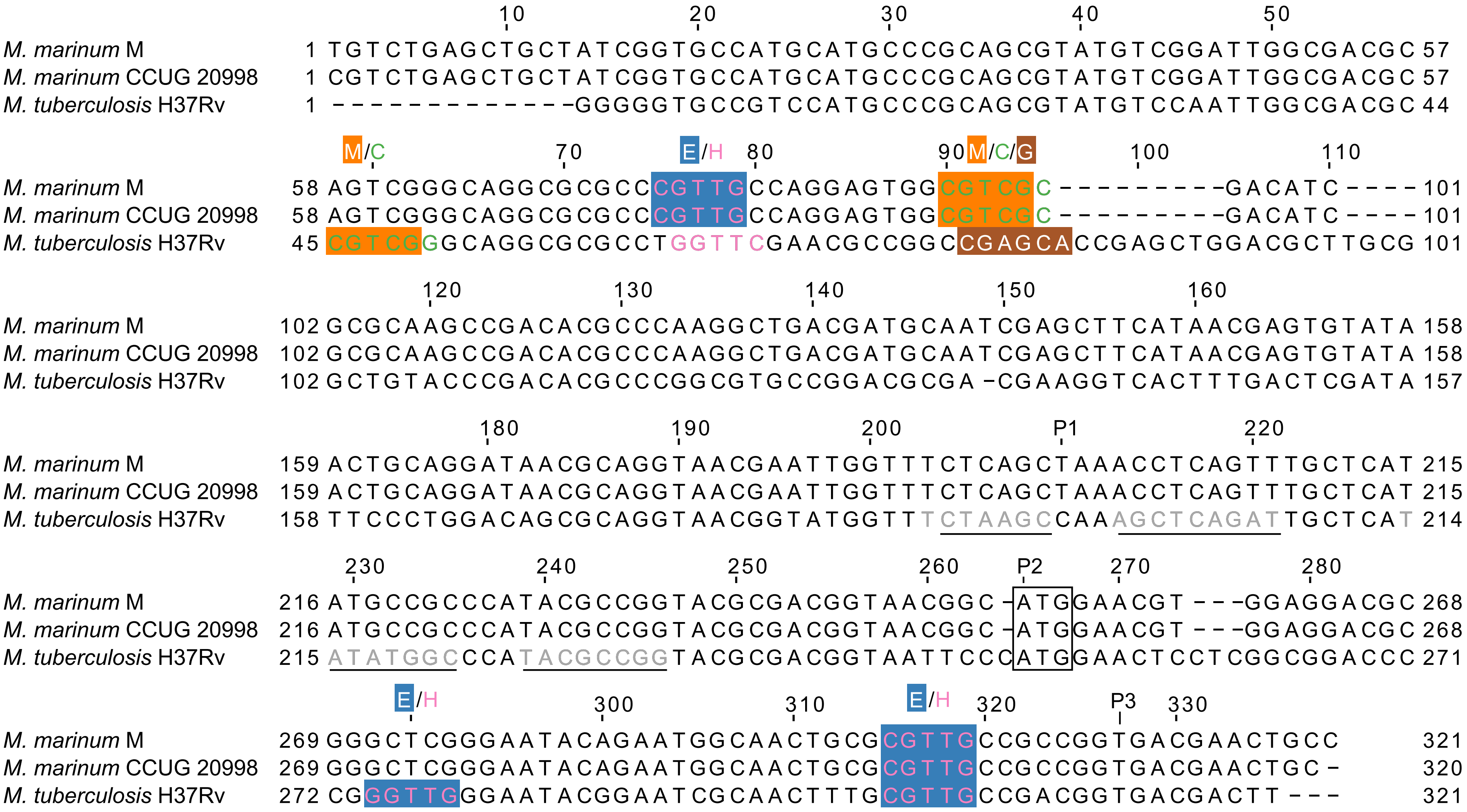

10 20 30 40 50
M. marinum M 1 TGTCTGAGCTGCTATCGGTGCCATGCATGCCCGCAGCGTATGTCGGATTGGCGACGC 57
M. marinum CCUG 20998 1 CGTCTGAGCTGCTATCGGTGCCATGCATGCCCGCAGCGTATGTCGGATTGGCGACGC 57
M. tuberculosis H37Rv 1 -------------GGGGGTGCCGTCCATGCCCGCAGCGTATGTCCAATTGGCGACGC 44
M/C 70 E/H 80 90 M/C/G 100 110
M. marinum M 58 AGTCGGGCAGGCGCGCCCGTTGCCAGGAGTGGCGTCGCC----------GACATC---- 101
M. marinum CCUG 20998 58 AGTCGGGCAGGCGCGCCCGTTGCCAGGAGTGGCGTCGCC----------GACATC---- 101
M. tuberculosis H37Rv 45 CGTCGGGCAGGCGCGCCTGGTTCGAACGCCGGCCGAGCACCGAGCTGGACGCTTGCG 101
120 130 140 150 160
M. marinum M 102 GCGCAAGCCGACACGCCCAAGGCTGACGATGCAATCGAGCTTCATAACGAGTGTATA 158
M. marinum CCUG 20998 102 GCGCAAGCCGACACGCCCAAGGCTGACGATGCAATCGAGCTTCATAACGAGTGTATA 158
M. tuberculosis H37Rv 102 GCTGTACCCGACACGCCCGGCGTGCCGGACGCGA-CGAAGGTCACTTTGACTCGATA 157
180 190 200 P1 220
M. marinum M 159 ACTGCAGGATAACGCAGGTAACGAATTGGTTTCTCAGCTAAACCTCAGTTTGCTCAT 215
M. marinum CCUG 20998 159 ACTGCAGGATAACGCAGGTAACGAATTGGTTTCTCAGCTAAACCTCAGTTTGCTCAT 215
M. tuberculosis H37Rv 158 TTCCCTGGACAGCGCAGGTAACGGTATGGTTTCTAAGCCAAAGCTCAGATTGCTCAT 214
230 240 250 260 P2 270 280
M. marinum M 216 ATGCCGCCCATACGCCGGTACGCGACGGTAACGGC-ATGGAACGT---GGAGGACGC 268
M. marinum CCUG 20998 216 ATGCCGCCCATACGCCGGTACGCGACGGTAACGGC-ATGGAACGT---GGAGGACGC 268
M. tuberculosis H37Rv 215 ATATGGCCCATACGCCGGTACGCGACGGTAATTCCCATGGAACTCCTCGGCGGACCC 271
E/H 300 310 E/H 320 P3 330
M. marinum M 269 GGGCTCGGGAATACAGAATGGCAACTGCGCGTTGCCGCCGGTGACGAACTGCC 321
M. marinum CCUG 20998 269 GGGCTCGGGAATACAGAATGGCAACTGCGCGTTGCCGCCGGTGACGAACTGC- 320
M. tuberculosis H37Rv 272 CGGGTTGGGAATACGGAATCGCAACTTTGCGTTGCCGACGGTGACGACTT--- 321

C

```
                                          rsbW
                              10        20        30        40
M. marinum M                1 GGCAGCGTGTTCGGCATCATGTTGACGGCTCGAAGGGTGGCCTCCGGC 48
M. marinum CCUG 20998       1 ----GCGTGTTCGGCATCATGTTGACGGCTCGAAGGGTGGCCTCCGGC 44
M. tuberculosis H37Rv       1 GGCAGTGTCTTCGGCATCACGTTGACCGCCCGACGGGCGGCATCCAGC 48

                                          sigF
                             50        60        70        80        90
M. marinum M               49 AGGTGACCTCCCGAACTGCCGGCGGTTCCTCTTCACGACCCAACGAAT 96
M. marinum CCUG 20998      45 AGGTGACCTCCCGAACTGCCGGCGGTTCCTCTTCACGACCCAACGAAT 92
M. tuberculosis H37Rv      49 AGGTGACGGCGCGCGCTGCCGGCGGTTCTGCATCGCGAGCTAACGAAT 96
```
