# Supplementary material for: Comparative Sigma Factor-mRNA Levels in Mycobacterium marinum under Stress Conditions and during Host Infection
Source: PLoS One. 2015 Oct 7;10(10):e0139823. doi: 10.1371/journal.pone.0139823 (PMC4596819; doi:10.1371/journal.pone.0139823)
Supplement: S1 Table — (PDF) [file pone.0139823.s009.pdf]

S1 Table. Significant (P < 0.05) $\log_2$-fold changes and their associated P-values relative to exponential phase.

	sigA	sigB	sigC	sigC_1	sigD	sigE	sigF	sigG	sigH	sigJ	sigK	sigL	sigM	sig0975	sig3276	sig3687	sig4487
Nitric oxide	1.1 (0.041)	NS (0.343)	2.5 (0.044)	1.6 (0.049)	0.3 (0.045)	NS (0.314)	NS (0.063)	NS (0.984)	NS (0.576)	NS (0.245)	0.2 (0.049)	NS (0.084)	1.4 (0.045)	< 0.1 (0.044)	1.2 (0.043)	NS (0.054)	0.4 (0.045)
Mitomycin C	0.1 (0.041)	NS (0.284)	2.2 (0.047)	0.4 (0.049)	NS (0.051)	0.5 (0.045)	NS (0.064)	NS (0.282)	NS (0.489)	NS (0.080)	0.1 (0.047)	1.0 (0.041)	0.1 (0.043)	0.4 (0.049)	0.2 (0.048)	0.2 (0.050)	0.1 (0.047)
Isoniazid	0.4 (0.043)	1.2 (0.041)	4.0 (0.039)	2.0 (0.045)	< 0.1 (0.050)	NS (0.638)	NS (0.055)	0.6 (0.042)	1.1 (0.049)	0.2 (0.043)	1.6 (0.044)	1.7 (0.041)	1.4 (0.045)	1.0 (0.047)	2.3 (0.045)	NS (0.458)	1.6 (0.041)
Osmotic	0.1 (0.049)	NS (0.179)	2.4 (0.044)	0.6 (0.050)	NS (0.217)	NS (0.359)	NS (0.192)	< 0.1 (0.046)	0.1 (0.045)	NS (0.130)	0.1 (0.045)	0.5 (0.048)	0.2 (0.044)	NS (0.057)	0.2 (0.046)	NS (0.100)	0.5 (0.043)
Oxidative	0.5 (0.044)	< 0.1 (0.043)	2.2 (0.044)	1.0 (0.048)	NS (0.066)	0.7 (0.043)	< 0.1 (0.049)	0.5 (0.047)	0.1 (0.044)	0.4 (0.045)	0.5 (0.047)	1.2 (0.048)	1.0 (0.047)	0.6 (0.048)	0.8 (0.049)	1.3 (0.049)	0.4 (0.045)
Acidic	0.7 (0.049)	NS (0.503)	1.4 (0.040)	1.3 (0.050)	0.9 (0.047)	< 0.1 (0.047)	0.4 (0.042)	0.3 (0.047)	NS (0.137)	NS (0.082)	0.7 (0.047)	0.4 (0.040)	1.8 (0.045)	0.8 (0.050)	1.4 (0.043)	1.2 (0.040)	0.8 (0.050)
Starvation	1.8 (0.047)	1.1 (0.050)	3.0 (0.045)	2.0 (0.048)	NS (0.466)	0.9 (0.050)	1.2 (0.046)	NS (0.096)	0.1 (0.044)	< 0.1 (0.149)	0.7 (0.046)	NS (0.108)	1.9 (0.043)	0.2 (0.049)	1.5 (0.045)	0.9 (0.047)	1.4 (0.046)
Heat	NS (0.834)	3.6 (0.046)	1.0 (0.045)	< 0.1 (0.042)	NS (0.663)	2.6 (0.042)	NS (0.416)	NS (0.825)	< 0.1 (0.042)	0.6 (0.046)	NS (0.475)	NS (0.072)	0.2 (0.045)	0.8 (0.037)	0.4 (0.046)	0.7 (0.041)	NS (0.165)
Cold	NS (0.145)	4.4 (0.048)	0.1 (0.043)	1.3 (0.049)	0.4 (0.049)	0.9 (0.040)	NS (0.552)	2.0 (0.042)	1.2 (0.036)	0.3 (0.048)	NS (0.083)	0.4 (0.040)	2.0 (0.049)	2.7 (0.047)	0.7 (0.042)	1.2 (0.045)	< 0.1 (0.037)
Microaerobic	3.5 (0.048)	< 0.1 (0.417)	5.4 (0.043)	4.8 (0.040)	3.3 (0.048)	1.9 (0.040)	3.2 (0.048)	2.2 (0.043)	1.9 (0.044)	2.2 (0.042)	4.1 (0.044)	3.0 (0.044)	2.7 (0.039)	3.1 (0.041)	4.4 (0.041)	1.7 (0.036)	4.8 (0.048)
Stationary (7H9)	0.9 (0.041)	0.7 (0.040)	4.1 (0.045)	2.8 (0.047)	1.7 (0.045)	0.9 (0.040)	1.4 (0.043)	0.5 (0.048)	0.7 (0.049)	NS (0.155)	1.2 (0.048)	0.0 (0.045)	0.9 (0.042)	0.2 (0.040)	1.5 (0.047)	2.5 (0.047)	0.8 (0.046)
Stationary (7H9)[#]	0.1 (0.047)	4.0 (0.043)	1.3 (0.048)	2.0 (0.043)	1.2 (0.045)	3.0 (0.043)	NS (0.078)	0.3 (0.037)	0.8 (0.043)	1.2 (0.025)	0.7 (0.043)	1.8 (0.047)	NS (0.062)	NS (0.400)	1.1 (0.046)	0.5 (0.048)	1.1 (0.038)

NS: not statistically significant. The numbers are given as: $\log_2$-fold changes (P-values). Some P-values are given as 0.050 due to round off. #: Relative to 7H10 2 days.
