# Supplementary material for: Comparative Sigma Factor-mRNA Levels in Mycobacterium marinum under Stress Conditions and during Host Infection
Source: PLoS One. 2015 Oct 7;10(10):e0139823. doi: 10.1371/journal.pone.0139823 (PMC4596819; doi:10.1371/journal.pone.0139823)
Supplement: S2 Table — (PDF) [file pone.0139823.s010.pdf]

S2 Table

M. marinum strains in this study.

Strain	Comment
M. marinum T CCUG 20998	From the Stock Center in Göteborg, Sweden
M. marinum CCUG^{rfp}	Derivative of *M. marinum* T CCUG 20998 with *rfp-hygR* introduced into the L5 *attB* site (see Pettersson et al., 2013; Mutoji and Ennis, 2012)
M. marinum DE4373	Derivative of *M. marinum* 1218R with *rfp-hygR* introduced into the L5 *attB* site (Mutoji and Ennis, 2012)
M. marinum DE4381	The *M. marinum* DE4381 strain (originally a derivative of *M. marinum* 1218S) is a variant of *M. marinum* 1218R that forms smooth colonies when grown on solid 7H10 medium, which is in contrast to *M. marinum* 1218R that forms rough colonies (Barker et al., 1997).
