# Supplementary material for: Comparative Sigma Factor-mRNA Levels in Mycobacterium marinum under Stress Conditions and during Host Infection
Source: PLoS One. 2015 Oct 7;10(10):e0139823. doi: 10.1371/journal.pone.0139823 (PMC4596819; doi:10.1371/journal.pone.0139823)
Supplement: S3 Table — (PDF) [file pone.0139823.s011.pdf]

S3 Table: Primers and probes for qRT-PCR analysis

Primers and probes	Sequence and labels[a]
UL051101 sigA F	CGACCAGGCCCGCA
UL051101 sigA R	ATGCGGCCCAGCTTGTT
ULP051110 sigA	6FAM-CCGGTGCACATGGT-MGBNFQ
FP1215-Mm_sigB_qRT_ fw	AGCCCTGCAGCGGACTT
FP1216-Mm_sigB_qRT_ rv	TTGAGCAACGCCGTCTTG
Mm_sigB-probe:	6FAM-TGCGCGTGTATCTG-MGBNFQ
FP1276-MM_sigC_qRT_fw	CGCATTCGAAGCATTCATCA
FP1277-MM_sigC_qRT_rv	CCGACAGGTAGGCCACAAAA
MMAR_sigC_probe	6FAM-CCACCCAGCACGACG-MGBNFQ
FP1274-MM_sigC1_qRT_fw	TCGGTCTAACGCTCGCACTT
FP1275-MM_sigC1_qRT_rv	CGGATCTGGTCGACAACCA
MMAR_sigC1_probe	6FAM-TTTGCGATCGCCCGTC-MGBNFQ
FP1213-Mm_sigD_qRT_ fw	CAGTGGCCTCTCAGCAGATG
FP1214-Mm_sigD_qRT_ rv	CCGTAATGGTGGCCAAACA
Mm_sigD-probe:	6FAM-TGTGGCACAGGAGGT-MGBNFQ
FP1163-MMsigE qRT fw	GACGAACTGCCGATGTCTGA
FP1164-MMsigE qRT rv	AAGGTGGTGATGGTGTGTTCCT
MMAR_4216sigE:	6FAM-CAGGGAAAAGCCG-MGBNFQ
FP1266-MM_sigG_qRT_fw	GAGACCCATGTGCCTTTTCAG
FP1415-MM_sigG_qRT_fw2[b]	GCGGGTGAGACCCATGTG
FP1267-MM_sigG_qRT_rv	GTGACACTCGGCCGTCAAC
MMAR_sigG_probe	6FAM-TGCATGTGCTGGATAT-MGBNFQ
FP1270-MM_sigH_qRT_fw	GCGTGGCTGTACCGGATT
FP1271-MM_sigH_qRT_rv	CGCTGCTTCTTGCGATAGCT
MMAR_sigH_probe	6FAM-TGACCAATACCTACATCAA-MGBNFQ
FP1268-MM_sigJ_qRT_fw	GCGTTCTACACGGCGTATCA
FP1269-MM_sigJ_qRT_rv	CCAGTGTGTAGGAGCCCAATTC
MMAR_sigJ_probe	6FAM-TGGCCTTGGTCAACG-MGBNFQ
FP1209-Mm_sigK_qRT_ fw	ACGCGTTCGCCATGTTCT
FP1210-Mm_sigK_qRT_ rv	CGTGCCACCAGTCCATACAC
Mm_sigK-probe:	6FAM-CGACCACACCTGTACAC-MGBNFQ
FP1211-Mm_sigL_qRT_ fw	CCTTGATGCAGGCGCTCTA
FP1212-Mm_sigL_qRT_ rv	ATTGCGTAGCGCCACAACA
Mm_sigL-probe:	6FAM-ACGAGCATGCGGCG-MGBNFQ
FP1272-MM_sigM_qRT_fw	CGGGCACAACGGCTCTT
FP1273-MM_sigM_qRT_rv	GACACGGCATTGCTTTTGG
MMAR_sigM_probe	6FAM-CGGTGCCTTGGGC-MGBNFQ
FP1319-sig0975fw-qRT	AGAATTGGCCTACGTCGTGAA
FP1320-sig0975rv-qRT	GGATGTGGGTCGCTGTGTTC
MM-sig0975pb	6FAM-ACATCGGTGCTATCC-MGBNFQ
FP1311-sig2297fw-qRT	CCAGCTTGGTTCGGGACTAC
FP1312-sig2297rv-qRT	CCATGGTGCGAGCGAAAT
MM-sig2997pb	6FAM-ACAGGCAGATGGTG-MGBNFQ
FP1313-sig3276fw-qRT	TTCCGGGTCGTGCTCAA
FP1314-sig3276rv-qRT	CACCGTATTGGCTTCCTTACG

Primers and probes	Sequence and labels[a]
MM-sig3276pb	6FAM-AGGTGCACACGCTGG-MGBNFQ
FP1315-sig3687fw-qRT	GGCTTTCGTGCTGCATGAC
FP1316-sig3687rv-qRT	CGAGGATGGGTGCGATCT
MM-sig3687pb	6FAM-TCTTCGCGGTCCCGTT-MGBNFQ
FP1317-sig4487fw-qRT	CCCGCGGTCCTGTTCTATC
FP1318-sig4487rv-qRT	ACGATTTCCAGCGTGATCACT
MM-sig4487pb	6FAM-CGGCGGGCAGTTG-MGBNFQ

a: 6-FAM = 6-carboxyfluorescein; MGBNFQ = Minor Groove Binder non-fluorescent quencher. b: Due to a point mutation in the *sigG* target sequence, a second forward primer was used for the DE 4373 strain.
