# Supplementary material for: Comparative Sigma Factor-mRNA Levels in Mycobacterium marinum under Stress Conditions and during Host Infection
Source: PLoS One. 2015 Oct 7;10(10):e0139823. doi: 10.1371/journal.pone.0139823 (PMC4596819; doi:10.1371/journal.pone.0139823)
Supplement: S4 Table — (PDF) [file pone.0139823.s012.pdf]

**S4 Table**

Colony count from infected organs as outlined in Materials and Methods.

Infection dose 2.8 x $10^2$ per fish and sacrificed 12 fish animals.

| Fish number | Organ | DE4373 output cfu/ organ |
|---|---|---|
| 1 | Liver | 3.5 x $10^7$ |
| | Kidney | 1.8 x $10^7$ |
| 2 | Liver | 4.7 x $10^5$ |
| | Kidney | 1.9 x $10^5$ |
| 3 | Liver | 7.7 x $10^4$ |
| | Kidney | 2.2 x $10^4$ |
| 4 | Liver | 1.1 x $10^5$ |
| | Kidney | None |
| 5 | Liver | 1.6 x $10^6$ |
| | Kidney | 2.2 x $10^6$ |
| 6 | Liver | 3.3 x $10^7$ |
| | Kidney | 6.7 x $10^6$ |
| 7 | Liver | 7.2 x $10^4$ |
| | Kidney | 8.5 x $10^4$ |
| 8 | Liver | 9.5 x $10^4$ |
| | Kidney | 3.2 x $10^4$ |
| 9 | Liver | 4.2 x $10^6$ |
| | Kidney | 1.2 x $10^6$ |
| 10 | Liver | 2.5 x $10^6$ |
| | Kidney | 3.7 x $10^4$ |
| 11 | Liver | 9.6 x $10^5$ |
| | Kidney | 6.6 x $10^5$ |
| 12 | Liver | None |
| | Kidney | None |
