# Supplementary material for: Comparative Sigma Factor-mRNA Levels in Mycobacterium marinum under Stress Conditions and during Host Infection
Source: PLoS One. 2015 Oct 7;10(10):e0139823. doi: 10.1371/journal.pone.0139823 (PMC4596819; doi:10.1371/journal.pone.0139823)
Supplement: S5 Table — (PDF) [file pone.0139823.s013.pdf]

S5 Table. Calculated p-values for the indicated times after stress induction relative to before induction.

		sigA	sigB	sigC	sigC_1	sigD	sigE	sigF	sigG	sigH	sigJ	sigK	sigL	sigM	sig0975	sig3276	sig3687	sig4487
Heat	1h	0.373	3.4E-5	0.005	0.177	0.435	0.001	0.276	0.884	0.243	0.253	0.066	0.010	0.964	0.388	0.346	0.602	0.422
	4h	0.663	5.0E-5	6.0E-4	0.032	0.156	1.2E-4	0.197	0.290	0.956	0.158	0.021	0.205	0.436	0.658	0.058	0.320	0.065
	6h	0.495	3.3E-5	8.6E-4	0.038	0.244	8.5E-5	0.301	0.130	0.593	0.116	0.018	0.608	0.323	0.355	0.047	0.562	0.075
	12h	0.345	3.6E-4	0.002	0.039	0.919	0.001	0.247	0.082	0.306	0.139	0.212	0.123	0.229	0.201	0.069	0.593	0.138
	24h	0.052	3.2E-5	0.009	0.229	0.227	2.7E-4	0.826	0.123	0.069	0.022	0.784	0.002	0.027	0.010	0.082	0.017	0.561
Cold	1h	0.031	3.5E-4	0.005	0.535	0.006	8.0E-4	0.077	0.238	0.342	0.316	0.974	0.004	0.020	0.003	0.013	0.031	0.304
	4h	0.172	1.1E-4	0.025	0.136	0.007	0.002	0.197	0.310	0.181	0.356	0.441	0.007	0.004	0.001	0.027	0.024	0.683
	6h	0.184	4.2E-5	0.018	0.110	0.005	0.002	0.175	0.157	0.087	0.220	0.344	0.003	0.002	5.5E-4	0.023	0.014	0.439
	12h	0.463	3.0E-5	0.080	0.029	0.007	0.003	0.686	0.016	0.024	0.134	0.063	0.007	0.001	6.1E-4	0.032	0.020	0.297
	24h	0.468	1.7E-5	0.072	0.015	0.007	0.004	0.889	0.004	0.008	0.055	0.036	0.015	0.002	6.2E-4	0.015	0.019	0.157
Microaerobic	0.375d	0.002	0.500	0.001	0.003	0.002	0.002	0.004	0.009	0.024	0.022	6.7E-4	0.020	0.013	0.032	0.003	0.307	0.002
	1d	0.001	0.063	0.003	0.001	0.002	0.003	0.002	0.014	0.041	0.031	4.6E-4	0.009	0.008	0.020	0.002	0.954	0.001
	3d	1.8E-4	0.588	2.3E-4	5.5E-4	3.2E-4	1.8E-4	6.0E-4	0.012	0.005	0.012	1.6E-4	0.001	0.003	5.1E-4	7.3E-4	0.065	4.0E-4
	6d	1.0E-4	0.334	6.7E-5	4.2E-4	7.0E-5	1.8E-4	3.0E-4	0.008	0.003	0.008	1.5E-4	2.2E-4	0.002	3.5E-4	4.6E-4	0.015	2.4E-4
	12d	6.7E-5	0.785	3.2E-5	3.7E-4	6.9E-5	1.8E-4	1.8E-4	0.004	0.002	0.006	1.6E-4	2.0E-4	0.001	2.9E-4	3.5E-4	0.005	1.9E-4

Not statistically significant changes are indicated by gray background shading.
